# HOTAIRM1 regulates neuronal differentiation by modulating NEUROGENIN 2 and the downstream neurogenic cascade

**DOI:** 10.1038/s41419-020-02738-w

**Published:** 2020-07-13

**Authors:** Jessica Rea, Valentina Menci, Paolo Tollis, Tiziana Santini, Alexandros Armaos, Maria Giovanna Garone, Federica Iberite, Andrea Cipriano, Gian Gaetano Tartaglia, Alessandro Rosa, Monica Ballarino, Pietro Laneve, Elisa Caffarelli

**Affiliations:** 1 Department of Biology and Biotechnology Charles Darwin, Sapienza University of Rome, Rome, Italy; 2 Center for Life Nano Science@Sapienza, Istituto Italiano di Tecnologia, Rome, Italy; 3 Centre for Genomic Regulation, The Barcelona Institute for Science and Technology, Barcelona, Spain; 4 Institució Catalana de Recerca i Estudis Avançats and Universitat Pompeu Fabra, Barcelona, Spain; 5 Institute of Molecular Biology and Pathology, National Research Council, Rome, Italy; 6 Present Address: Nouscom, Rome, Italy; 7 Present Address: The BioRobotics Institute and Department of Excellence in Robotics & AI, Scuola Superiore Sant’Anna, Pisa, Italy; 8 Present Address: Department of Obstetrics & Gynecology, Stanford University, Stanford, CA USA

**Keywords:** Proteomics, Differentiation, Gene silencing, Long non-coding RNAs, Neurogenesis

## Abstract

Neuronal differentiation is a timely and spatially regulated process, relying on precisely orchestrated gene expression control. The sequential activation/repression of genes driving cell fate specification is achieved by complex regulatory networks, where transcription factors and noncoding RNAs work in a coordinated manner. Herein, we identify the long noncoding RNA HOTAIRM1 (HOXA Transcript Antisense RNA, Myeloid-Specific 1) as a new player in neuronal differentiation. We demonstrate that the neuronal-enriched HOTAIRM1 isoform epigenetically controls the expression of the proneural transcription factor *NEUROGENIN 2* that is key to neuronal fate commitment and critical for brain development. We also show that HOTAIRM1 activity impacts on NEUROGENIN 2 downstream regulatory cascade, thus contributing to the achievement of proper neuronal differentiation timing. Finally, we identify the RNA-binding proteins HNRNPK and FUS as regulators of HOTAIRM1 biogenesis and metabolism. Our findings uncover a new regulatory layer underlying *NEUROGENIN 2* transitory expression in neuronal differentiation and reveal a previously unidentified function for the neuronal-induced long noncoding RNA HOTAIRM1.

## Introduction

Mammalian brain neurons derive from self-renewing neural stem cells (NSCs) whose differentiation occurs in two distinct steps: the initial production of neuronal precursors (NPs), already committed to a neuronal fate, and the following formation of differentiated cells that acquire and maintain their identity^1^.

Proneural genes (PGs) are key regulators of neurogenic differentiation. They encode transcription factors (TFs) of the basic helix–loop–helix (bHLH) class, mainly acting as transcriptional activators. In vertebrates, PGs are first expressed in NSCs. However, their activity is carried out only later, when they reach high levels of expression, and gives rise to asymmetrically dividing NPs that are restricted to a neuronal fate. This is achieved through activation of neuronal-differentiation gene cascades, inhibition of glial cell fates and regulation of cell cycle^2^. The proneural TF NEUROGENIN 2 (NEUROG2) is a tightly regulated master gene in neuronal differentiation. In NSCs, it is controlled by the TF ETV5^3^ and the bHLH repressors HES. In particular, the oscillatory and inverse correlated expression of *HES* genes and *NEUROG2* impedes its neurogenic activity^4,5^, contributing to the maintenance of NSCs^6–8^. *NEUROG2* is, instead, upregulated in NPs by the TFs PAX6^9^ and SOX2, which acts in combination with the long noncoding RNA (lncRNA) RMST^10^. *NEUROG2* sustained and persistent expression instructs the neuronal differentiation programme by activating downstream targets that induce the expression of differentiation-related genes^11^ and the repression of the stemness factor PAX6^12^.

Once the neuronal programme has been triggered, *NEUROG2* expression must be turned off to allow the differentiation to proceed and to guarantee the maintenance of neuronal cell identity. We previously demonstrated that the Microprocessor complex, together with the RNA-binding protein TDP-43, is implicated in NEUROG2 gene silencing by inducing the degradation of its mRNA^13^. However, the mechanisms modulating *NEUROG2* repression in differentiating neurons are still poorly characterised.

LncRNAs are powerful regulators of gene expression^14,15^. Notably, 40% of human-annotated lncRNAs are expressed in the brain where, through multiple mechanisms^16^, they impinge on every step of neurodevelopment, from differentiation to synaptogenesis^14,17–19^.

Herein, we assign a new function to the lncRNA HOTAIRM1 (HOXA Transcript Antisense RNA, Myeloid-Specific 1), a transcript previously implicated, as a *cis*-acting factor, in the regulation of *HOXA* gene cluster^20,21^ and in myeloid maturation^22^. This study presents the first evidence of the involvement of HOTAIRM1 in neuronal differentiation. By exploiting different in vitro model systems, we demonstrate that, in the nucleus, the neuronal HOTAIRM1 isoform controls the transitory expression of *NEUROG2*, which impacts on the downstream neurogenic regulatory cascade.

## Materials and methods

### Cell cultures and manipulations

SH-SY5Y and NB4 cells were obtained from ATCC and DSMZ, respectively. Induced pluripotent stem cells (iPSCs) were derived and maintained as in ref. ^23^. Details about cell culture, differentiation and transfection protocols are available in SI Materials and Methods. LNA GapmeRs and siRNAs are listed in Table S1.

### FISH and immunofluorescence

SH-SY5Y cells and iPSC-derived human MNs were cultured on pre-coated glass coverslips and then fixed in 4% paraformaldehyde/PBS. FISH experiments were performed as described in ref. ^24^ and in SI Materials and Methods.

### RNA extraction and analysis

RNA expression analyses were performed through quantitative or semiquantitative real-time PCR (qRT-PCR or RT-PCR) on cDNA synthetised by Takara PrimeScript RT Reagent Kit (RR037A, Takara-bio).

### Cell fractionation

Three-day RA-treated SH-SY5Y cells were fractionated according to the Ambion PARIS Kit (AM1921, Life Technologies). After RNA extraction, equal volumes of cytoplasmic or nuclear RNA were retro-transcribed and analysed by qRT-PCR. Normalisations were based on the total amount of RNA.

### Immunoblotting

Whole-cell or nucleus/cytoplasm fractionated protein extracts were prepared from SH-SY5Y cells in RIPA buffer and processed as indicated in SI Materials and Methods.

### RNA immunoprecipitation (RIP) assay

Nuclear RIP assay was carried out as in ref. ^13^. Details and specific antibodies are reported in SI Materials and Methods.

### Chromatin immunoprecipitation (ChIP) assay

Chromatin extracts were prepared from 1 × 10^6^ proliferating or differentiating SH-SY5Y cells (eventually knocked-down for nHOTAIRM1) after cross-linking in 1% formaldehyde. Immunoprecipitation was performed using the MAGnify Chromatin Immunoprecipitation System kit (492024, Invitrogen). Details in SI Materials and Methods.

### Cross-linking immunoprecipitation (CLIP) assay

Totally, 75 × 10^6^ SH-SY5Y cells were grown with RA for 6 days, UV cross-linked at 4000 × 100 µj/cm^2^ and collected in NP-40 lysis buffer. Protocols for immunoprecipitation and RNA analysis are detailed in SI Materials and Methods.

### RNA antisense purification-mass spectrometry (RAP-MS) assay

RAP-MS assay was performed on total cell extracts, as described in ref. ^25^. Probe design, cell manipulation, experimental procedure and RNA and protein analyses are detailed in SI Materials and Methods.

### *cat*RAPID analyses

The *cat*RAPID algorithm^26^ predicts the interaction potential of a protein and RNA pair. Interaction propensity is calculated from the primary structure alone^26^ and has been experimentally shown to estimate the binding affinity^27,28^. As demonstrated also in this work, *cat*RAPID algorithm is able to separate interacting vs. non-interacting pairs with an area under the receiving operating characteristics (ROC) curve of 0.76 in >500,000 experimentally validated interactions^29^. Details of specific calculations for nHOTAIRM1 and U1 interactions are reported in SI Materials and Methods.

### Statistical analyses

Histograms show the mean ± SEM from 2 to 4 biological replicates. *N* is indicated in Figure Legends. Errors were calculated from relative quantities and then opportunely propagated; statistical significance was determined by two-tailed paired Student's *t* test. A *p* value (*p*) < 0.05 was considered as significant.

Analyses of variance were done using two-way ANOVA followed by post hoc Tukey′s test (see SI Materials and Methods).

### Oligonucleotides

Sequences of oligonucleotides are listed in Table S2.

## Results

### HOTAIRM1 and *NEUROG2* expression are inversely correlated during neuronal differentiation

**Fig. 1:**
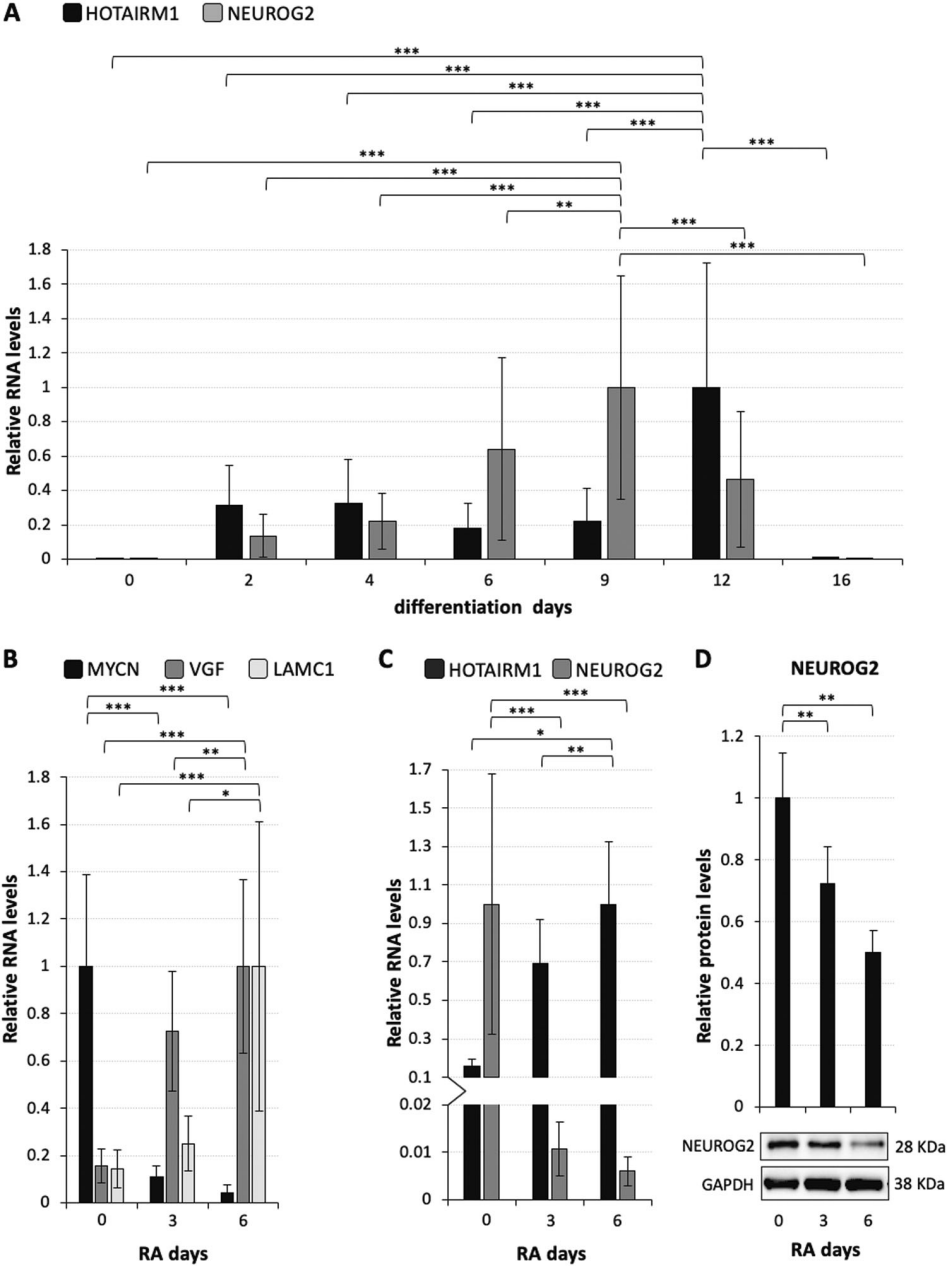
HOTAIRM1 and NEUROG2 expression are inversely correlated during neuronal differentiation. **a** qRT-PCR analysis of HOTAIRM1 and *NEUROG2* expression along differentiation of iPSCs into ventral spinal cord lineages. Differentiation days are reported on the *x*-axis. Expression levels are relative to *ATP5O* and expression peaks are set as 1. *N* = 1, 2 or 3, depending on the sample. **P ≤ 0.0021, ***P ≤ 0.0002. **b** qRT-PCR analysis of mRNAs coding for MYCN (proliferative marker), VGF (neuropeptide precursor) and LAMC1 (neurite-promoting factor) along neuronal differentiation of SH-SY5Y cells. Days of RA treatment are reported on the *x*-axis. Expression levels are relative to *GAPDH*, expression peaks are set as 1. *N* = 3, *P ≤ 0.05, **P ≤ 0.01, ***P ≤ 0.001. **c** qRT-PCR analysis of HOTAIRM1 and *NEUROG2* expression along neuronal differentiation of SH-SY5Y cells. Broken axis-histogram allows to appreciate low values. Details as in (**b**). *N* = 2 or 3, depending on the target. *P ≤ 0.05, **P ≤ 0.01, ***P ≤ 0.001. **d** Immunoblot analysis of NEUROG2 along neuronal differentiation of SH-SY5Y cells. Days of RA treatment are reported below each lane. In the histogram, NEUROG2 levels are relative to GAPDH. *N* = 3, **P ≤ 0.01.

RNA-Seq analysis revealed that HOTAIRM1 is significantly upregulated in human neurons derived from iPSCs^30^. By querying genotype-tissue expression (GTEx) Analysis Release V7 (dbGaP Accession phs000424.v7.p2) we found that, among thirteen brain tissues, HOTAIRM1 is exclusively expressed in the spinal cord (cervical c-1) (Fig. S1A). These lines of evidence suggested a possible role for HOTAIRM1 in the regulation of spinal neuron differentiation. To address this issue, we exploited human iPSCs induced to differentiate into ventral spinal cord lineages, which include about 30% of motoneurons (MNs)^31^ (Fig. S1B). In this context, we profiled the expression of HOTAIRM1 (Fig. 1a), whose genomic localisation is shown in Fig. S1C, together with that of the neuronal marker NEUROG2. HOTAIRM1 is induced at early stages (day 2), is stably accumulated until day 9 (NPs, marked by NEUROG2 (FIG. S1B)), reaches its maximum level in differentiating neurons (day 12), to strongly diminish in post-mitotic cells (day 16, marked by CHAT and ISLET (Fig. S1B)). Notably, at the time-window from day 9 to 12 an inverse correlation between HOTAIRM1 and NEUROG2 is evident and it was statistically corroborated by an overall significant two-way ANOVA analysis followed by Tukey′s multiple comparisons test (alpha = 0.05) (Dataset 1). This result suggests a possible role for the lncRNA as a negative regulator of *NEUROG2* expression in that time-window.

To investigate this potential function, we used the human neuroblastoma derived SH-SY5Y cells, considered to be NPs^32^. Upon retinoic acid (RA) treatment, they recapitulate the transition between NPs and differentiating neurons^33^, which occurs between day 9 and 12 of iPSC differentiation. RA administration produces a homogeneous population of mature neuron-like cells^34,35^, which express a variety of neuronal markers^34,36^. Figure 1b shows a typical differentiation time course of SH-SY5Y cells treated with RA for 3 and 6 days. The levels of differentiation markers *VGF*^37^ and *LAMC1*^38^ were gradually induced up to 6 days, whereas the proliferative marker *MYCN*^39^ dropped down already at 3 days. *NEUROG2* and HOTAIRM1 profiles showed their inversely correlated expression (Fig. 1c). The levels of HOTAIRM1 increased up to 6 days of RA treatment (about sixfold increase compared to control). Conversely, the levels of *NEUROG2* were highest in undifferentiated SH-SY5Y cells according to their NP state^33^, drastically decreased at 3 days of RA-treatment and further reduced at later times (day 6). Accordingly, also the levels of NEUROG2 protein decreased (Fig. 1d). The expression profiles of HOTAIRM1 and *NEUROG2* indicate that, both in differentiating iPSCs (Fig. 1a) and in RA-treated SH-SY5Y cells (Fig. 1c), the induction of HOTAIRM1 corresponds to a significant downregulation of *NEUROG2*.

### HOTAIRM1 is localised in both nuclear and cytoplasmic compartments of in vitro-derived neurons

**Fig. 2:**
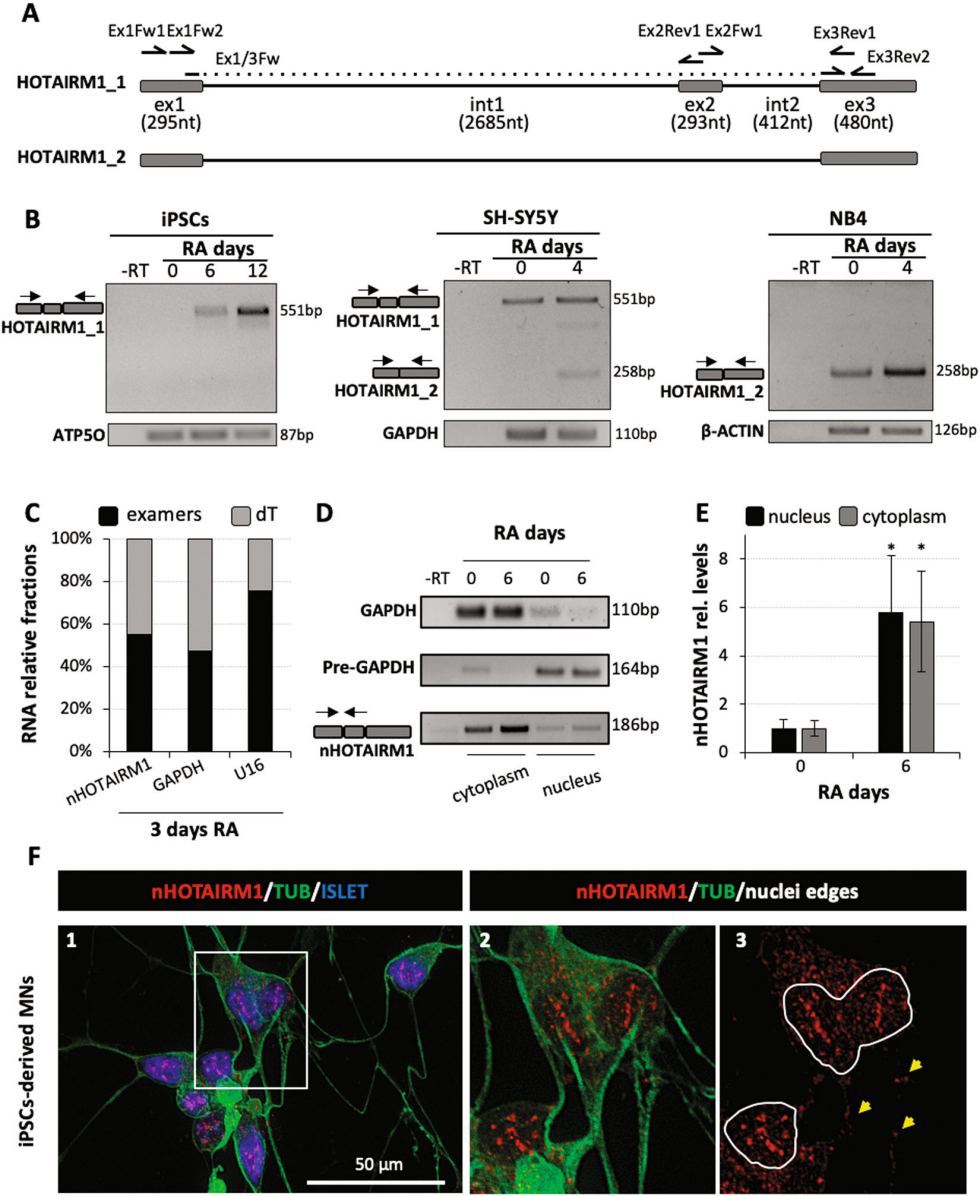
Molecular characterisation of HOTAIRM1. **a** Schematic representation of HOTAIRM1 transcript variants (HOTAIRM1_1 and HOTAIRM1_2) according to UCSC Genome browser. Exons (ex) are indicated as boxes, introns (int) as a continuous line. Lengths (nucleotides, nt) are reported between brackets. Oligonucleotides used for semi-qRT-PCR and qRT-PCR amplifications are indicated. **b** Semi-qRT-PCR analysis of HOTAIRM1 isoforms (described in panel (**a**)) in iPS (left panel), SH-SY5Y (middle panel) and NB4 (right panel) differentiating cells. Differentiation days are indicated above each lane. -RT samples were used as negative controls and *ATP5O*, *GAPDH* or *β-ACTIN* as internal standards. Amplifications were performed through primers Ex1Fw1 and Ex3Rev1, indicated in panel (**a**). Expected amplicon sizes are indicated on the right. **c** qRT-PCR analysis of HOTAIRM1_1 (nHOTAIRM1) polyadenylated/un-polyadenylated fractions (expressed as % of total transcript levels) in 3-day RA-treated SH-SY5Y cells. Reverse transcription was performed by random examers (examers) or dT oligonucleotides (dT). *GAPDH* and U16 snRNA were used as representative polyadenylated or un-polyadenylated species, respectively. Amplifications were performed through primers Ex2Fw1 and Ex3Rev1. *N* = 1. **d** Semi-qRT-PCR analysis of nHOTAIRM1 subcellular localisation in nuclear and cytoplasmic fractions of undifferentiated (day 0) and 6-day RA-treated (day 6) SH-SY5Y cells. Fractionation efficiency was assessed by *GAPDH* and *pre-GAPDH* analysis in the nucleus and cytoplasm, respectively. -RT samples were used as negative controls. nHOTAIRM1 amplifications were performed through primers Ex1Fw2 and Ex2Rev1. Expected amplicon sizes are indicated on the right. **e** qRT-PCR analysis of nHOTAIRM1 in the nucleus and the cytoplasm of differentiating SH-SY5Y cells. Expression at day 0 was set as 1. Normalisation was performed on total RNA. *N* = 3, *P ≤ 0.05. **f** nHOTAIRM1 localisation in FACS-purified MNs. Panel 1: RNA FISH analysis combined with immunofluorescence in MNs purified by FACS from differentiated human iPSCs; nHOTAIRM1, TUBULIN III (TUB) and ISLET 1/2 (ISLET) are stained in red, green and blue, respectively. Panel 2: digital magnification of the square insert of panel 1, without DAPI staining. Panel 3: the same picture as in panel 2 with nuclei edges; yellow arrowheads point to nHOTAIRM1 FISH signals in the neurites.

NCBI's Reference Sequence Database (RefSeq) in the UCSC Genome Browser (GRCh38/hg38) reports two HOTAIRM1 isoforms: the transcript variant 1 (HOTAIRM1_1; NR_038366.1), comprising three exons, and the transcript variant 2 (HOTAIRM1_2; NR_038367.1), lacking exon 2 (Fig. 2a). In RA-mediated myeloid differentiation, HOTAIRM1 was described as a unique transcript corresponding to HOTAIRM1_2^20^, whereas a spliced isoform (HOTAIRM1_1) and an unspliced transcript were detected in an axial development system^21^.

By semi-qRT-PCR, we analysed HOTAIRM1 variants both in differentiating iPS and SH-SY5Y cells and, as control, in NB4 promyelocytic leukaemia cells induced to myeloid differentiation^20^. Figure 2b shows that only HOTAIRM1_1 was detected in differentiating iPSCs (left panel) and that it represents the major isoform in RA-treated SH-SY5Y cells (middle panel). HOTAIRM1_2 was, instead, the only transcript observed in RA-treated NB4 cells (right panel and ref. ^20^). These findings indicate that, while HOTAIRM1_2 is the myelopoietic-enriched transcript, HOTAIRM1_1 represents the neuronal-enriched isoform. We addressed the following molecular, functional and mechanistic studies on this isoform, referred hereafter as nHOTAIRM1 (neuronal HOTAIRM1).

As HOTAIRM1_2^20^, nHOTAIRM1 is a polyadenylated transcript (Fig. 2c). Its subcellular localisation was explored through both biochemical fractionation and RNA fluorescence in situ hybridisation (FISH) analyses. Subcellular fractionations indicated that nHOTAIRM1 was present both in the nucleus and cytoplasm of proliferating (day 0) and differentiating (day 6) SH-SY5Y cells, the cytoplasmic component being more abundant (Fig. 2d). RA-mediated upregulation of nHOTAIRM1 was comparable in both compartments (about sixfold increase; Fig. 2e).

FISH analysis was performed in fluorescence-activated cell sorting (FACS)-purified MNs derived from iPSCs upon 12 days of differentiation (Fig. 2f) and, as control, in RNase-treated MNs (Fig. S2). According to biochemical data, this analysis confirmed the double localisation of the lncRNA in post-mitotic neurons (Fig. 2f, panels 1 and 2). Notably, cytoplasmic nHOTAIRM1 was localised both in the soma and neurites of MNs, suggesting it might participate in local RNA regulation (Fig. 2f, panel 3). Altogether, these results indicate that nHOTAIRM1 has a dual cellular localisation and, in principle, it might exert different functions in different cell compartments.

### Nuclear nHOTAIRM1 regulates *NEUROG2* and the downstream neurogenic cascade

**Fig. 3:**
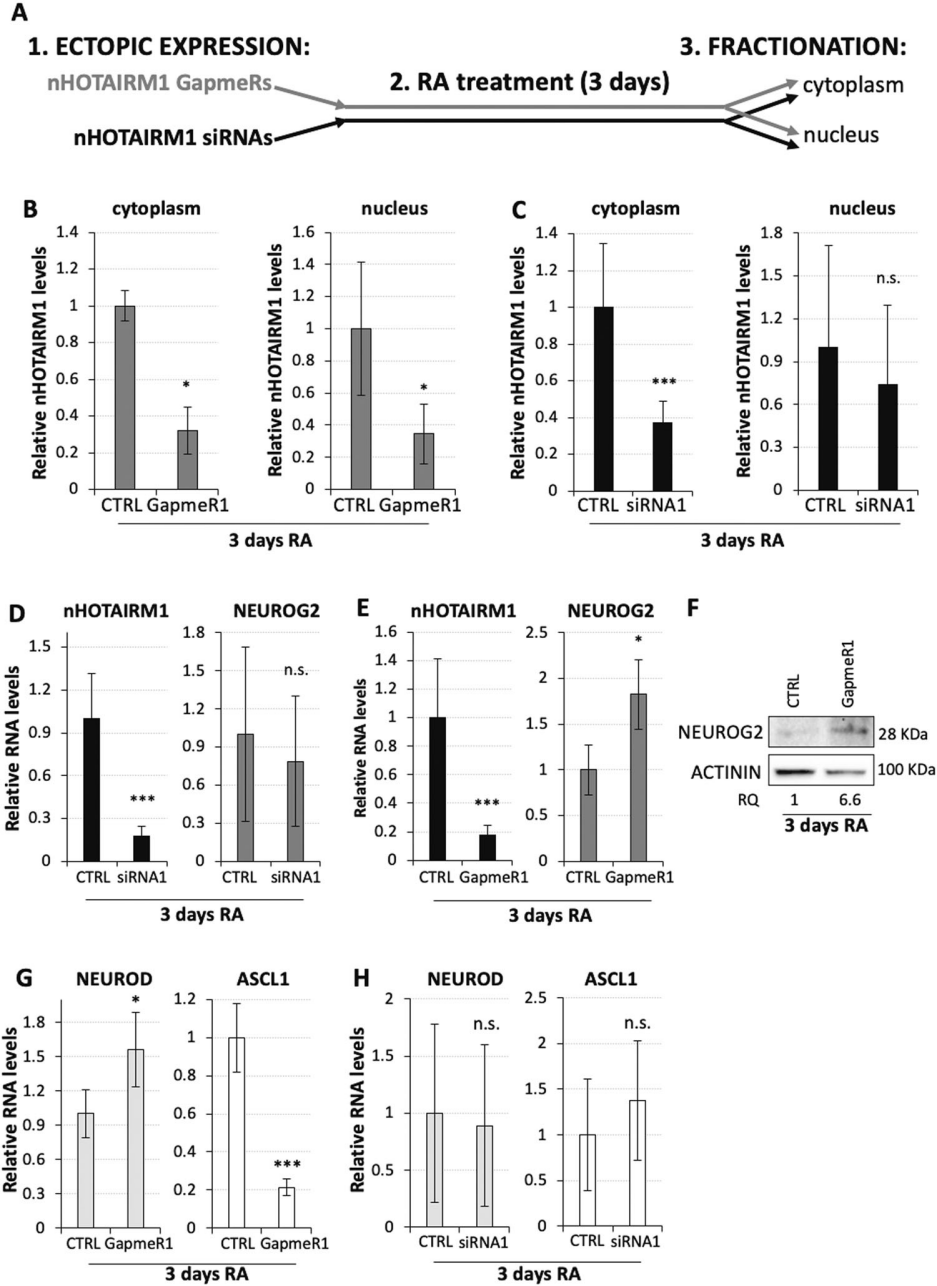
nHOTAIRM1 loss-of-function affects the expression of *NEUROG2* and its target genes. **a** Schematic representation of the experimental design. nHOTAIRM1 LNA GapmeRs or siRNAs are ectopically expressed in differentiating SH-SY5Y cells, then separated into nuclear and cytoplasmic fractions. **b** qRT-PCR analyses of nHOTAIRM1 expression in the cytoplasm (left panel) and the nucleus (right panel) of 3-day RA treated SH-SY5Y cells, transfected with LNA GapmeRs (GapmeR1). Expression was set as 1 in scrambled-transfected cells (CTRL). Normalisation was performed on total RNA. *N* = 3, *P ≤ 0.05. **c** qRT-PCR analysis of nHOTAIRM1 expression in the cytoplasm (left panel) and the nucleus (right panel) of 3-day RA treated SH-SY5Y cells, transfected with siRNAs (siRNA1). Details as in (**b**). *N* = 4, ***P ≤ 0.001. **d** qRT-PCR analyses of nHOTAIRM1 (left histogram) and *NEUROG2* (right histogram) expression upon siRNA-mediated nHOTAIRM1 knockdown (siRNA1) in 3-day RA treated SH-SY5Y cells. Scrambled siRNAs were used as negative control (CTRL) and set as 1. Data are relative to *GAPDH*. *N* = 3, ***P ≤ 0.010. **e** qRT-PCR analyses of nHOTAIRM1 (left histogram) and *NEUROG2* (right histogram) expression upon GapmeR-mediated nHOTAIRM1 knock-down (GapmeR1) in 3-day RA treated SH-SY5Y cells. Scrambled GapmeRs were used as negative control (CTRL) and set as 1. Details as in (**d**). *N* = 3 or 4, depending on the target. *P ≤ 0.05, ***P ≤ 0.001. **f** Immunoblot analysis of NEUROG2 protein levels upon GapmeR1-mediated nHOTAIRM1 knockdown in 3-day RA treated SH-SY5Y cells. Scrambled GapmeRs were used as negative control (CTRL) and ACTININ as internal standard. Relative quantity (RQ) was expressed with respect to control cells set as 1 and reported below each lane. *N* = 1. **g** qRT-PCR analyses of *NEUROD* (left histogram) and *ASCL1* (right histogram) expression upon GapmeR1-mediated nHOTAIRM1 knockdown in 3-day RA treated SH-SY5Y cells. Details as in (**d**). *N* = 4, *P ≤ 0.05, ***P ≤ 0.001. **h** qRT-PCR analyses of *NEUROD* (left histogram) and *ASCL1* (right histogram) expression upon siRNA1-mediated nHOTAIRM1 knockdown in 3-day RA treated SH-SY5Y cells. Details as in (**d**). *N* = 2.

Based on the inverse correlation between nHOTAIRM1 and *NEUROG2* expression (Fig. 1a, c) we ascertained, by a loss-of-function approach, whether nHOTAIRM1 may act as a negative regulator of *NEUROG2* during neuronal differentiation. To differentially knockdown nuclear and cytoplasmic nHOTAIRM1 species, we designed two sets of antisense oligonucleotides: LNA GapmeRs, more effective for nuclear targets, and siRNAs, that function preferentially in the cytoplasm^40,41^ (Fig. 3a).

RA-treated SH-SY5Y cells were transfected with siRNAs or LNA GapmeRs, and RNA purified from nuclear and cytoplasmic fractions (Figs. S3A, B) was analysed by qRT-PCR. LNA GapmeR1 caused a ~60% reduction of both cytoplasmic and nuclear nHOTAIRM1 (Fig. 3b), whereas siRNAs exclusively knocked-down cytoplasmic nHOTAIRM1 (by about 60%) (Fig. 3c). Therefore, the parallel use of the two sets of antisense oligonucleotides allowed us to discriminate between nuclear and cytoplasmic nHOTAIRM1 functions.

We evaluated *NEUROG2* levels in 3-day RA-treated cells upon siRNA- or GapmeR-mediated nHOTAIRM1 silencing. No any modulation of *NEUROG2* was observed when the expression of cytoplasmic nHOTAIRM1 was silenced by siRNAs targeting two different lncRNA regions (Figs. 3d, S4A). Differently, an about twofold increase of *NEUROG2* mRNA levels was achieved upon 80% reduction of nHOTAIRM1 by different LNA GapmeRs directed against the neuronal isoform (Figs. 3e, S4B). Pursuant to *NEUROG2* mRNA upregulation, a sixfold increase of NEUROG2 protein was detected (Fig. 3f). These data demonstrated that only the nuclear nHOTAIRM1 negatively controls *NEUROG2* expression. As a specificity-control, we tested whether HOTAIRM1 may functionally interact with *MYCN*, which, similarly to *NEUROG2*, showed an inverse expression profile with the lncRNA during differentiation (Fig. 1b). Interestingly, we found that HOTAIRM1 GapmeR-mediated knockdown did not impact *MYCN* expression in differentiating SH-SY5Y cells (Fig. S4C).

To realise the full implications of the aberrant increase of NEUROG2 protein, we evaluated the levels of two downstream targets in the neurogenic cascade, the proneural factors *NEUROD* and *ASCL1*. NEUROG2 activates *NEUROD*^11^ and represses *ASCL1*^42^ expression during differentiation. LNA GapmeR-mediated knockdown of nHOTAIRM1 (GapmeR1, Fig. 3e) caused an increase of *NEUROD* mRNA levels by ~50% and a decrease of *ASCL1* mRNA by ~80% (Fig. 3g). No any variation of these NEUROG2 targets was observed when nHOTAIRM1 silencing was performed with siRNAs (siRNA1, Fig. 3h). These results, confirmed by the use of additional siRNAs (siRNA2, Fig. S4D) and LNA GapmeRs (GapmeR2, Fig. S4E), entail that nuclear nHOTAIRM1 regulates *NEUROG2* expression and its downstream neurogenic cascade during in vitro neuronal differentiation.

### Nuclear nHOTAIRM1 is required for the epigenetic regulation of *NEUROG2* expression

**Fig. 4:**
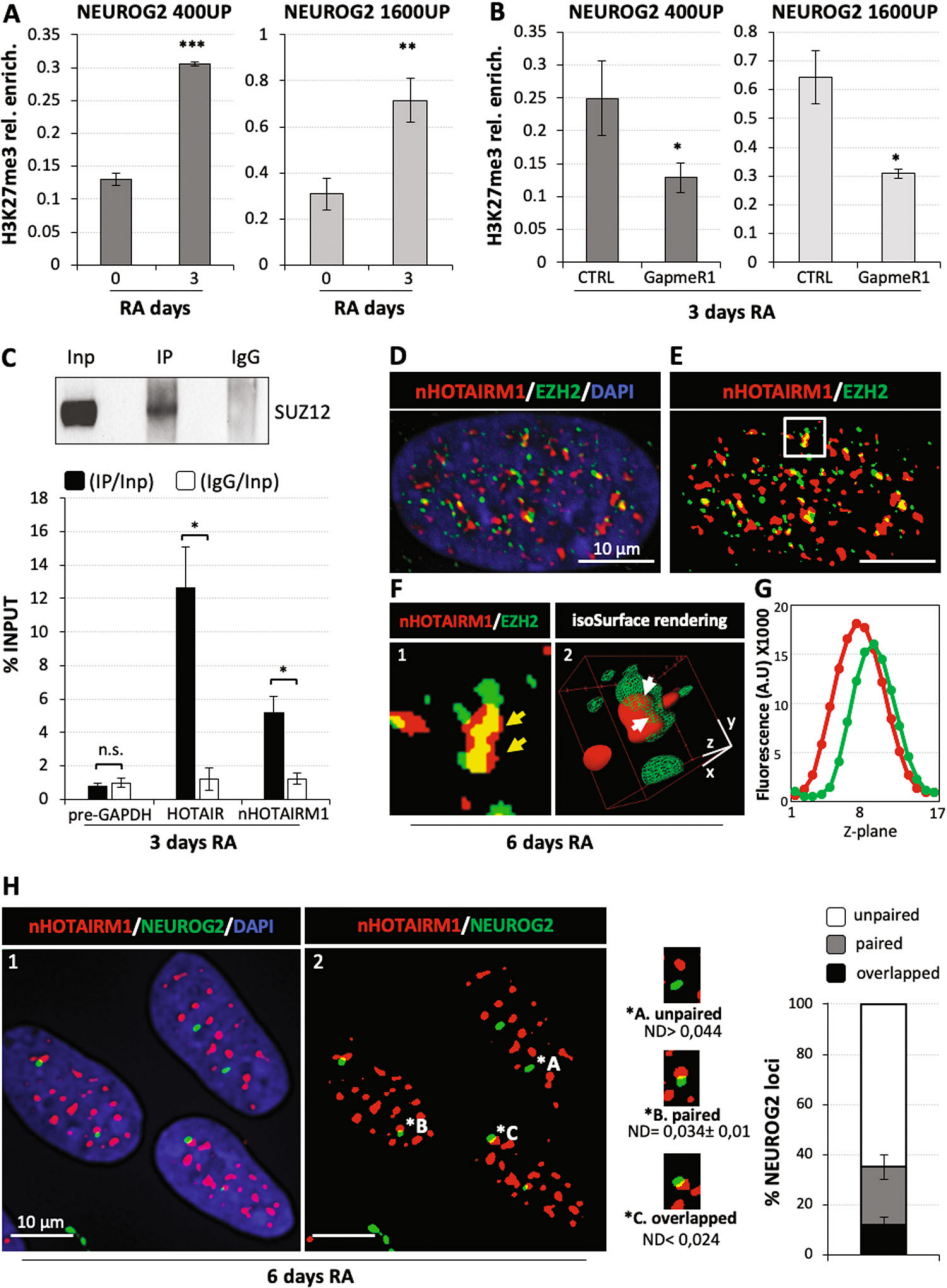
nHOTAIRM1 epigenetically regulates *NEUROG2* through PRC2. **a** H3K27me3 occupancy on regions 400 bp (400UP, left panel) or 1600 bp (1600UP, right panel) upstream of *NEUROG2* TSS (see Fig. S5A) in SH-SY5Y differentiating cells (day 3), compared to untreated cells (day 0). An intergenic region (2000 bp upstream of the HPRT1 TSS; see Fig. S5B) was used to normalise the two conditions. Enrichments are expressed as percentage relative to Input. *N* = 4, **P ≤ 0.01, ***P ≤ 0.001. **b** H3K27me3 occupancy on *NEUROG2* upstream regions upon nHOTAIRM1 knockdown (GapmeR1) compared to control-transfected cells (CTRL), in 3-day RA-treated SH-SY5Y cells. Details as in (**a**). *P ≤ 0.05. **c** SUZ12-mediated RNA immunoprecipitation assay in 3-day RA-treated SH-SY5Y cells. Upper panel: immunoblot analysis of SUZ12 in Input (Inp), immunoprecipitated (IP) and IgG (IgG) protein fractions. Lower panel: RNA enrichment over Input, in IP and IgG protein fractions. Data are expressed as percentage of Input. *Pre-GAPDH* and HOTAIR enrichments were used as negative and positive controls, respectively. *N* = 3, *P ≤ 0.05. **d** Combined RNA FISH and IF analyses showing the spatial correlation between nHOTAIRM1 and EZH2 in 6-day RA-treated SH-SY5Y cells: representative fluorescence image (Z-projection of confocal stacks) indicating the localisation of nHOTAIRM1 RNA (red signals) and EZH2 protein (green signals) in the nucleus (DAPI, blue staining). **e** RNA FISH/IF staining presented in panel (**d**) converted to a binary image, to highlight the overlapping signals (yellow). **f** Magnification of the square insert in (**e**) showing the colocalized regions (yellow arrows) in a 2D representation (panel 1) and in a 3D volume rendering (isosurface) (panel 2). In the latter panel, white arrows show the local points where nHOTAIRM1 RNA and PRC2 signals are overlapped. **g** Plot of Z-stack intensity distribution for colocalized nHOTAIRM1/EZH2 signals reported in panel (**f**). The curves show the fluorescence intensity profile in each channel (A.U. = arbitrary units) along Z-confocal planes (Z-step = 200 nm). The correspondence of the distributions indicates the 3D spatial correlation of the signals. The 3D-colocalization between nHOTAIRM1 and EZH2 was detected in 26.32% ± 1.5 of nHOTAIRM1 RNA FISH signals analysed in 39 nuclei (mean ± SEM, *N* = 3). **h** RNA/DNA FISH for nHOTAIRM1 RNA (red signals) and *NEUROG2* locus (green signals) in 6-day RA treated SH-SY5Y (panel 1). Signal conversion into binary image is shown in panel 2: magnifications of three representative conditions (unpaired, paired or overlapped spots) are reported aside. Histogram on the right shows a quantitative analysis, in one representative experiment, of *NEUROG2* loci unpaired, paired or overlapped to nHOTAIRM1 spots, expressed as the mean ± SD percentage. Scale bar = 10 μm.

Nuclear lncRNAs often function as molecular scaffolds mediating epigenetic modifications in cis and/or in trans^16^. It has been shown that HOTAIRM1 recruits both polycomb repressive complex 2 (PRC2) and trithorax complex during axial development^21^. Based on these observations, we asked whether nHOTAIRM1 affected the epigenetic status of NEUROG2 promoter. First, we examined whether *NEUROG2* expression was epigenetically silenced during neuronal differentiation. To this aim, we evaluated the activity of PRC2, which promotes the epigenetic silencing by H3K27 trimethylation^43–46^, on *NEUROG2* promoter^4^ in undifferentiated vs. RA-treated SH-SY5Y cells. Chromatin immunoprecipitation (ChIP) assay, performed on two selected promoter regions (Fig. S5A), revealed a twofold increase of H3K27me3 repressive mark at 3 days of differentiation (Fig. 4a), which correlates with the observed drastic reduction of *NEUROG2* expression (Fig. 1c). Increased H3K27me3 enrichment was not observed on the promoter of the HPRT1 control gene (Fig. S5B, C).

To test the involvement of nHOTAIRM1 in such epigenetic silencing, we performed ChIP analysis in RA-treated cells upon nHOTAIRM1 knockdown by LNA GapmeRs. Depletion of nHOTAIRM1 by ~80% (Fig. 3e) caused a significant reduction of H3K27me3 deposition on both regions of *NEUROG2* promoter (Fig. 4b), while it did not affect the control gene (Fig. S5D). This result demonstrates that nHOTAIRM1 is implicated in the control of the epigenetic status of *NEUROG2* during neuronal differentiation, by favouring H3K27 trimethylation.

To corroborate this evidence, we monitored the ability of the lncRNA to associate with PRC2 by RNA immunoprecipitation assay (RIP assay) and by RNA FISH combined with immunofluorescence analysis (RNA FISH/IF). Immunoprecipitation of the PRC2 component SUZ12, performed in nuclear extracts from differentiating SH-SY5Y cells (Fig. 4c, upper panel), revealed the interaction between SUZ12 and nHOTAIRM1 (Fig. 4c, lower panel). In line with this result, a combined FISH/IF approach highlighted the partial overlap between nHOTAIRM1 RNA and PRC2 signals (Fig. 4d, e, magnified in Fig. 4f, panel 1). In particular, ~25% of the nHOTAIRM1 signals showed spatial proximity with the PRC2 catalytic component EZH2 at the same focal planes, indicating 3D-colocalization of the signals, as shown by isosurface rendering (Fig. 4f, panel 2) and fluorescence plot (Fig. 4g).

Supporting the role of nHOTAIRM1 as a direct interactor of *NEUROG2* locus, RNA/DNA FISH revealed the spatial proximity between the lncRNA and *NEUROG2* genomic region in differentiating SH-SY5Y cells (Fig. 4h). As shown in the histogram aside, ~35% of spots corresponding to *NEUROG2 loci* (green signals) are in paired or overlapped configuration to nHOTAIRM1 spots (red signals).

We conclude that nHOTAIRM1 is functionally required for recruiting the epigenetic machinery that catalyses H3K27me3 modification on *NEUROG2* locus, during neuronal differentiation.

### Identification of nHOTAIRM1 protein interactors

**Fig. 5:**
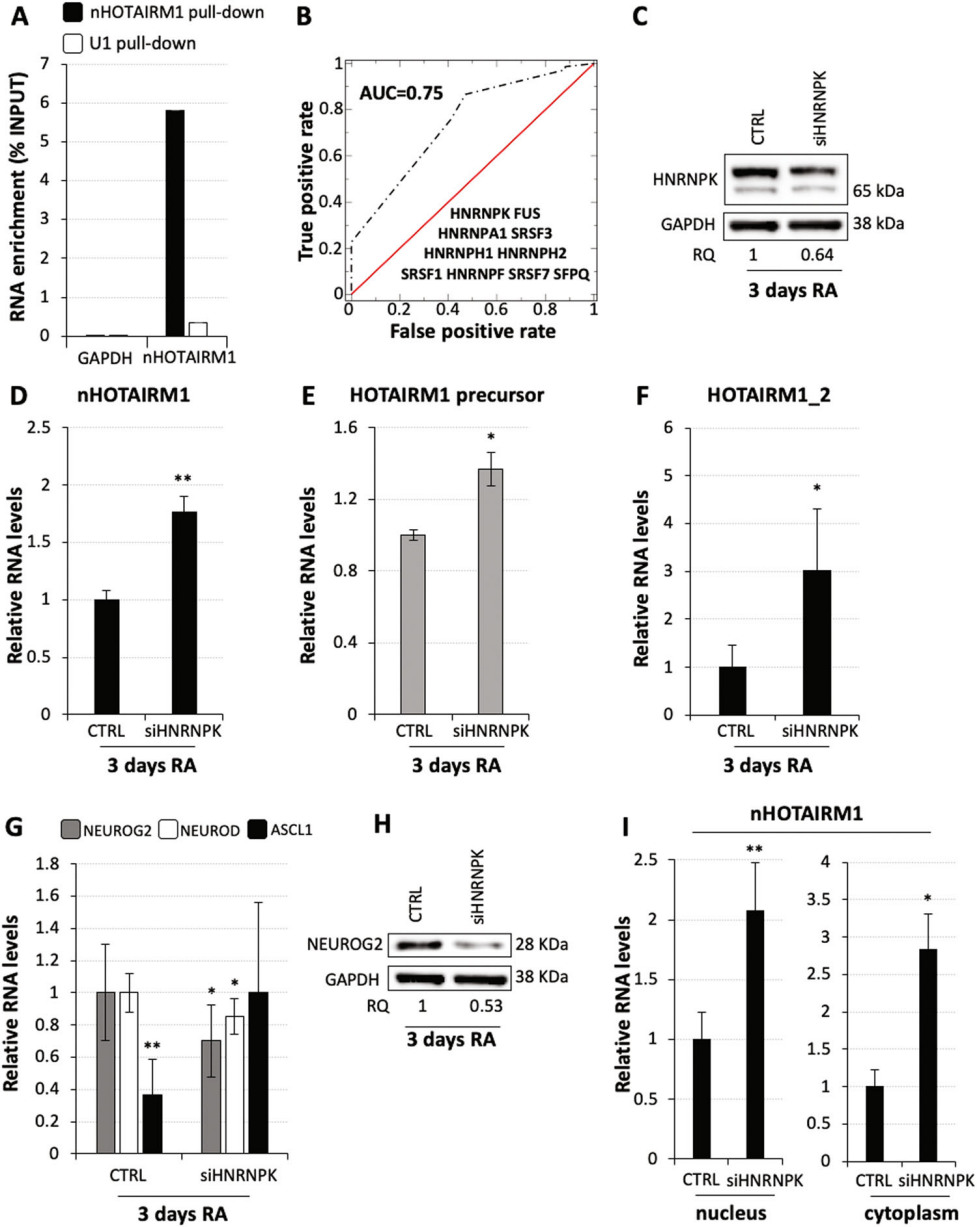
HNRNPK is a regulator of nHOTAIRM1 metabolism. **a** qRT-PCR analysis of nHOTAIRM1 from RAP assay in differentiating MNs. RNA enrichments over Input, in nHOTAIRM1 or U1 pull-down fractions are reported. Data expressed as percentage of Input, *N* = 1. **b** Consensus between predicted and experimentally validated interactors of nHOTAIRM1. We report a ROC (receiving operating characteristics) with AUC (area under the curve) of 0.75 between experimental and computational results. We consider as positives all the proteins with *cat*RAPID interaction propensities >2 and RAP-MS NSAF scores >0.01. **c** Immunoblot analysis of HNRNPK protein upon siRNA-mediated HNRNPK knockdown in 3-day RA-treated SH-SY5Y cells. Scrambled siRNAs were used as negative control (CTRL). Relative quantity (RQ) is expressed with respect to control cells set as 1 and reported below each lane. HNRNPK levels were quantified relative to GAPDH. *N* = 1. **d** qRT-PCR analysis of nHOTAIRM1 expression upon siRNA-mediated HNRNPK knockdown in 3-day RA-treated SH-SY5Y cells. Scrambled siRNAs were used as negative control (CTRL). Data are relative to *GAPDH*. *N* = 4, **P ≤ 0.01. **e** qRT-PCR analysis of HOTAIRM1 primary transcript (precursor) upon siRNA-mediated HNRNPK knockdown in 3-day RA-treated SH-SY5Y cells. Details as in (**d**). *N* = 3, *P ≤ 0.05. **f** qRT-PCR analysis of HOTAIRM1_2 (myeloid transcript) upon siRNA-mediated HNRNPK knockdown in 3-day RA-treated SH-SY5Y cells. Amplifications were performed through primers Ex1/3Fw and Ex3Rev2 (see Fig. 2a). Details as in (**d**). *P ≤ 0.05. **g** qRT-PCR analyses of *NEUROG2* and its downstream pathway genes (*NEUROD* and *ASCL1*) upon siRNA-mediated HNRNPK knockdown in 3-day RA-treated SH-SY5Y cells. Expression peaks are set as 1. *N* = 2 or 4, depending on the target. *P ≤ 0.05, **P ≤ 0.01. **h** Immunoblot analysis of NEUROG2 protein levels upon siRNA-mediated HNRNPK knockdown in 3-day RA-treated SH-SY5Y cells. Details and statistics as in (**c**). **i** qRT-PCR analyses of nHOTAIRM1 in nuclear (left histogram) or cytoplasmic (right histogram) fractions from 3-day RA-treated SH-SY5Y cells, upon HNRNPK knockdown. Expression in scramble-transfected cells was set as 1 (CTRL). Normalisation was performed on total RNA. *N* = 4, *P ≤ 0.05, **P ≤ 0.01.

RNAs are co-transcriptionally assembled into functional ribonucleoprotein particles^47^ whose protein moiety is informative on both RNA metabolism and activity. To identify nHOTAIRM1 direct protein partners in living cells, we exploited RNA antisense purification (Fig. 5a) coupled with mass spectrometry (RAP-MS) analysis^25^. The experiment was performed in differentiating spinal MNs—expressing high levels of nHOTAIRM1 (Fig. S6A)—derived from human iPSCs through a fast method^48,49^. To assess whether RAP worked properly, it was applied in parallel to U1 snRNA (Fig. S6B), whose protein interactors are well-known^50^. Indeed, U1 RAP-MS analysis revealed strong enrichment of U1snRNP specific components, as SNRNP70, SNRPA and SNRPC and the Sm proteins SNRPB, SNRPD1, SNRPD2, SNRPD3, SNRPE and SNRPGP15 (Dataset 2).

To produce a list of nHOTAIRM1 high-confidence protein interactors, we combined the experimentally identified hits with the in silico calculations generated through the *cat*RAPID algorithm (Materials and methods)^51,52^ that eliminate any experimental bias between the sample and the control. Analysing about 3500 potential protein–RNA interactions, we found strong agreement between *cat*RAPID predictions and RAP-MS experiments, with an area under the ROC curve of 0.75. In Fig. 5b, we report the proteins with strong *cat*RAPID interaction propensities (scores > 2) and detected in RAP-MS with an normalised spectrum abundance factor (NSAF) score >0.01 (Dataset 2). nHOTAIRM1 direct interactors include SR-splicing factors, as SFPQ^53^, SRSF1, SRSF3, SRSF7^54^ and heterogeneous nuclear ribonucleoproteins (HNRNPs)^55^ as HNRNPA1, HNRNPH1, HNRNPH2, HNRNPF, HNRNPK and FUS^56^. Directly comparing *cat*RAPID interaction propensities for nHOTAIRM1 and U1, we identified HNRNPK and FUS as the hits with the largest binding preference for nHOTAIRM1 (Dataset 2, Column I). Thus, following the experimental and computational prioritisation of HNRNPK and FUS, we decided to further investigate their roles in nHOTAIRM1 function and metabolism.

### The RNA-binding proteins HNRNPK and FUS control nHOTAIRM1 levels

The HNRNPK protein, whose levels are downregulated during in vitro neuronal differentiation (Fig. S7A and ref. ^57^), is involved in several cellular pathways such as transcription, splicing and translation^58^. To understand the significance of its interaction with nHOTAIRM1, we knocked down HNRNPK expression in differentiating SH-SY5Y cells and evaluated the lncRNA levels. A decrease of ~30% of *HNRNPK* mRNA (Fig. S7B) and protein (Fig. 5c) levels caused an increase of both mature (about 80%, Fig. 5d) and primary (about 40%, Fig. 5e) nHOTAIRM1 transcripts. In line with HNRNPK activity as a transcriptional repressor^58^ this may be explained, at least in part, by lncRNA transcriptional activation. Remarkably, a significant accumulation of the myeloid-enriched variant HOTAIRM1_2, which is normally absent or expressed at low levels in neuronal systems (Fig. 2b), was also observed upon HNRNPK depletion (about 300% increase, Fig. 5f). Altogether, these results suggest that HNRNPK participates in the control of nHOTAIRM1 metabolism, at both the transcriptional and splicing levels.

Next, we asked whether the HNRNPK-mediated nHOTAIRM1 gain-of-function (Fig. 5d) may impinge on NEUROG2 pathway. We found that HNRNPK silencing in differentiating SH-SY5Y cells caused a 30% decrease of *NEUROG2* mRNA (Fig. 5g) and a ~50% reduction of NEUROG2 protein (Fig. 5h), which resulted in the alteration of *NEUROD* and *ASCL1* expression (Fig. 5g). This is consistent with an increased level of nHOTAIRM1 in the nucleus as verified by cell fractionation (Fig. 5i). Notably, the effect of nHOTAIRM1 gain-of-function is complementary to the one obtained through its silencing (Fig. 3e, g), leading to the conclusion that both loss- and gain-of-function of nuclear nHOTAIRM1 impinge on NEUROG2 cascade.

**Fig. 6:**
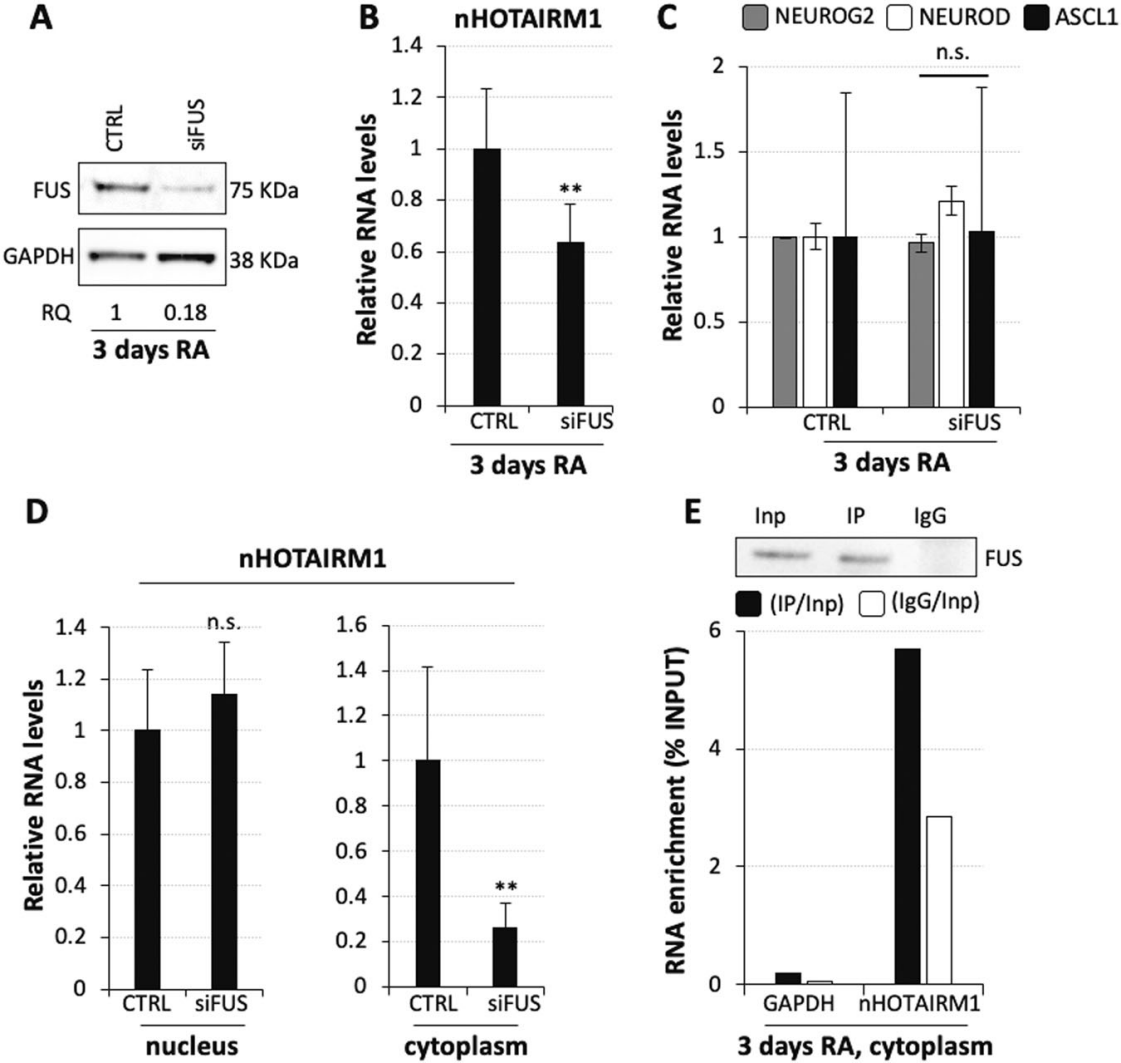
FUS regulates the levels of cytoplasmic nHOTAIRM1. **a** Immunoblot analysis of FUS protein upon siRNA-mediated FUS knockdown in 3-day RA-treated SH-SY5Y cells. Scrambled siRNAs were used as negative control (CTRL). Relative quantity (RQ) was expressed with respect to control cells set as 1 and reported below each lane. FUS levels were quantified relative to GAPDH. *N* = 1. **b** qRT-PCR analysis of nHOTAIRM1 expression upon siRNA-mediated FUS knockdown in 3-day RA-treated SH-SY5Y cells. Expression in scramble-transfected cells (CTRL) is set as 1. Data are expressed relative to *GAPDH*. *N* = 3, **P ≤ 0.01. **c** qRT-PCR analyses of *NEUROG2*, *NEUROD* and *ASCL1* upon siRNA-mediated FUS knockdown in 3-day RA-treated SH-SY5Y cells. Details as in (**b**). *N* = 2 or 3, depending on the target. **d** qRT-PCR analyses of nHOTAIRM1 in nuclear (left histogram) or cytoplasmic (right histogram) fractions from RA-treated SH-SY5Y cells, upon FUS knockdown. Expression in scramble transfected cells (CTRL) was set as 1. Normalisation was performed on total RNA. *N* = 2 or 3, depending on the sample. **P ≤ 0.01. **e** CLIP assay for FUS in the cytoplasmic fraction of 3-day RA-treated SH-SY5Y cells. Upper panel: immunoblot analysis of FUS in Input (Inp) extract, immunoprecipitated (IP) and IgG (IgG) protein fractions. Lower panel: qRT-PCR analysis of RNA enrichment over Input, in IP and IgG fractions. Data are expressed as Input percentage. *GAPDH* was used as a negative control. *N* = 1.

The second ranked nHOTAIRM1 interactor is FUS, an RNA-binding protein downregulated during neuronal differentiation (Fig. S8A and ref. ^59^), which plays a relevant role in brain development and neurodegeneration^60–62^. FUS knockdown experiments in differentiating SH-SY5Y cells produced an 80% reduction of FUS mRNA and protein (Figs. S8B, Fig. 6a). This caused a decrease of nHOTAIRM1 levels by ~40% (Fig. 6b). Nevertheless, FUS knockdown did not cause any alteration of *NEUROG2*, *NEUROD* and *ASCL1* mRNAs (Fig. 6c). This is in line with the finding that *FUS* silencing affected only the cytoplasmic nHOTAIRM1 transcript (Fig. 6d). Accordingly, CLIP assay revealed FUS/nHOTAIRM1 interaction in the cytoplasm (Fig. 6e). These data unveil FUS as a direct regulator of nHOTAIRM1 abundance in the cytoplasm and confirm that nHOTAIRM1 cytoplasmic transcript does not participate in the control of the NEUROG2-mediated pathway.

## Discussion

**Fig. 7:**
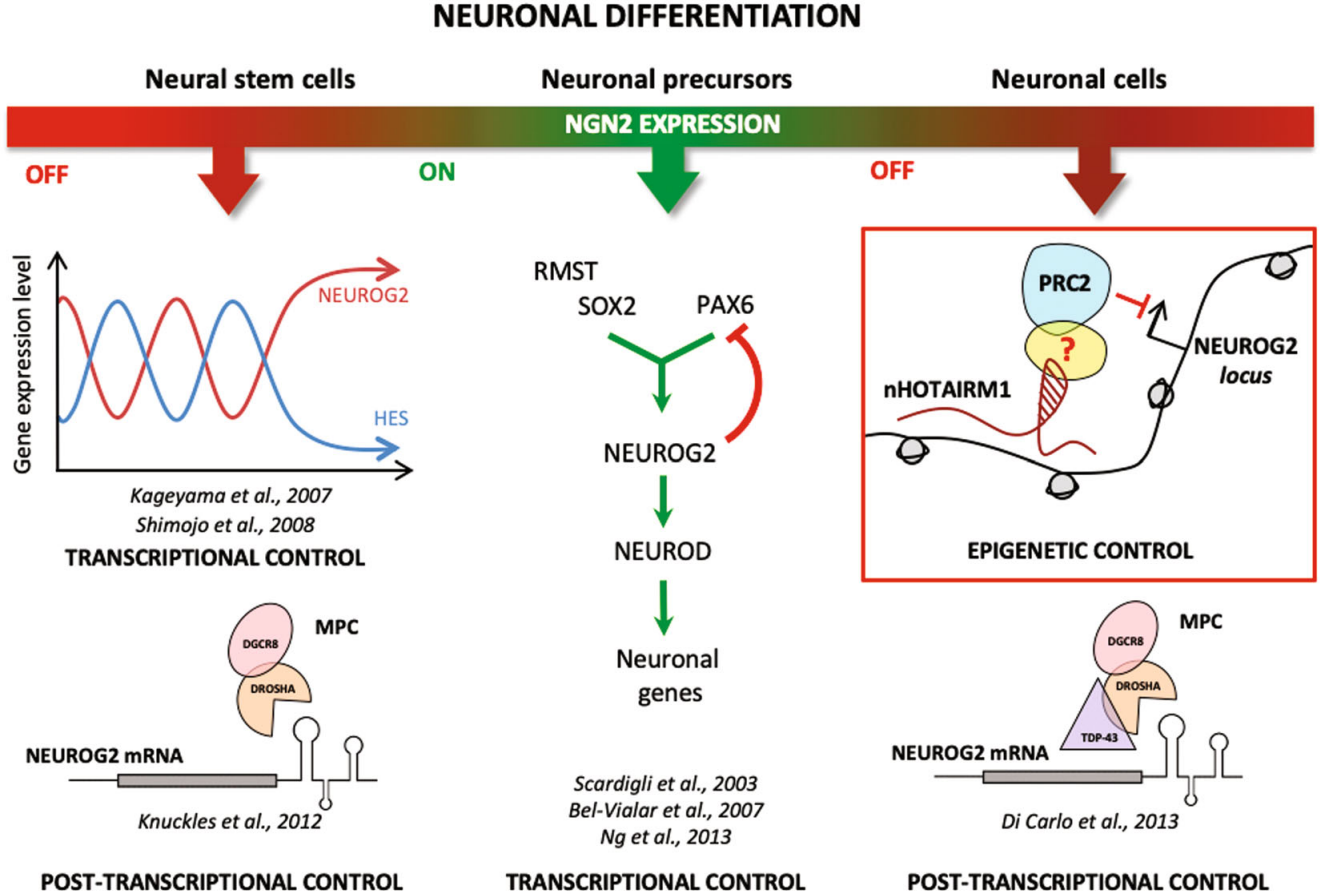
A novel layer of *NEUROG2* regulation. Epigenetic, transcriptional and post-transcriptional regulatory mechanisms converging on *NEUROG2* contribute to its transitory expression during neuronal differentiation. The role of nHOTAIRM1 as a scaffold that coordinates the recruitment of the repressive epigenetic machinery PRC2 to *NEUROG2* gene promoter in differentiating neurons is highlighted (red box).

Fine regulation of *NEUROG2* during differentiation is crucial for the entire neurogenesis. Even subtle alterations of its expression are detrimental for brain development and organisation^63^. High levels of NEUROG2 in NPs are required to instruct neuronal differentiation, by inducing neurogenic gene cascades and by repressing genes responsible for cell proliferation. Instead, low levels of the protein are essential in differentiating neurons to allow the temporal progression of neurogenesis and to maintain their differentiated state. Therefore, NEUROG2 abundance is an important determinant controlled by multiple regulatory mechanisms that, acting at proper differentiation times, secure the neuronal cell fate. So far, both transcriptional^5,9^ and post-transcriptional mechanisms^13,64^ have been described for *NEUROG2* repression during neuronal differentiation (Fig. 7). Here, we discover a novel epigenetic layer of regulation for *NEUROG2* and highlight the contribution of the lncRNA nHOTAIRM1 to this control.

To perform molecular, functional, and mechanistic studies, we exploited two neuronal differentiation models that recapitulate the neuronal differentiation in vitro, the iPS and the SH-SY5Y cells. Using these systems we: (i) profiled the expression of *NEUROG2* and nHOTAIRM1 during neuronal differentiation, (ii) underscored their inverse correlation in the transition between NPs and differentiating neurons, (iii) characterised the neuronal isoform of HOTAIRM1, unveiling nuclear and cytoplasmic transcripts and (iv) demonstrated that the nuclear lncRNA is required for *NEUROG2* repression at the proper differentiation time. Importantly, perturbations of the lncRNA levels impinge on at least two direct targets of NEUROG2, namely NEUROD, whose activation promotes neuronal differentiation^11^, and ASCL1, whose repression switches the cell fate from proliferation to differentiation during neural development^41^. This demonstrates that, by controlling *NEUROG2* expression, nHOTAIRM1 drives the downstream gene cascade.

At the mechanistic level, we found that nuclear nHOTAIRM1 functions as a *trans*-acting molecule that mediates the epigenetic control of *NEUROG2* expression by recruiting PRC2, which deposits the H3K27me3 repressive mark^42,44,45^ on *NEUROG2* promoter.

The interaction between lncRNAs and PRC2 has been extensively debated. The binding of PRC2 to the RNA may be promiscuous or specific, depending on the experimental conditions and the cellular context^65–69^. In living cells, several parameters conferring specificity—such as the occurrence of bridging factors—should be considered. Even though RIP and FISH assays evidenced the association of nHOTAIRM1 with PRC2 in differentiating neurons, none of these methods can indicate whether the interaction is direct or not. The *cat*RAPID algorithm^51,52^ predicted that neither EZH2 nor SUZ12 have strong binding propensities to nHOTAIRM1 (scores < 1.5, Dataset 2), suggesting that the interaction of the lncRNA with PRC2 is mediated by bridging proteins, which will be investigated in the future. Consistently, PRC2 components were not found among nHOTAIRM1 direct interactors by RAP-MS.

Being implicated in a relevant biological pathway, the levels of nHOTAIRM1 must be finely regulated. This is suggested by its expression profile, which is modulated along iPSC differentiation (Fig. 1a), and by the findings that both decrease and increase of nHOTAIRM1 abundance affect *NEUROG2* cascade (Figs. 3e, g, 5d, g). This study identifies two direct protein interactors, the multifunctional RNA-binding proteins HNRNPK and FUS, contributing to the control of nHOTAIRM1 expression levels. HNRNPK may affect nHOTAIRM1 metabolism at the transcriptional and splicing levels. Splicing control is particularly relevant since it establishes the ratio between the neuronal and myeloid transcripts, which are expressed in a cell-specific manner during the differentiation processes (Fig. 2b and refs. ^20,22^). Therefore, HNRNPK may be included in the regulatory cascade governing neuronal differentiation (Fig. S9). Differently, the other interactor FUS cannot be integrated in NEUROG2 neurogenic pathway, since it affects the level of the cytoplasmic nHOTAIRM1 possibly conferring stability to the transcript and mediating additional functions of the lncRNA.

Besides HNRNPK and FUS, other factors were unveiled as direct nHOTAIRM1 interactors. Some of them, as HNRNP H2 and F are nuclear regulators of alternative splicing^70^, whereas others, such as HNRNPA1^71^ and the SR proteins SFPQ, SRSF1, SRSF3, SRSF7, shuttle between the nucleus and the cytoplasm^72^. This suggests a wide spectrum of additional activities for nHOTAIRM1, both as a splicing factor hijacker in the nucleus, as a translational regulator in the cytoplasm and as an export adaptor for the proteins that shuttle between the two compartments^72^. Therefore, we cannot exclude the possibility that nHOTAIRM1 might contribute to neuronal differentiation through additional pathways, besides the NEUROG2 cascade.

In conclusion, this study allowed us to add a new tile to the mosaic of *NEUROG2* regulation (Fig. 7) and revealed a novel role for the lncRNA nHOTAIRM1 in shaping the outcome of neurogenesis.

## Supplementary information

Supplementary information

Supplementary figure 1

Supplementary figure 2

Supplementary figure 3

Supplementary figure 4

Supplementary figure 5

Supplementary figure 6

Supplementary figure 7

Supplementary figure 8

Supplementary figure 9

Supplementary table s1

Supplementary table s2

Supplementary dataset 1

Supplementary dataset 2
