# Supplementary material for: HOTAIRM1 regulates neuronal differentiation by modulating NEUROGENIN 2 and the downstream neurogenic cascade
Source: Cell Death Dis. 2020 Jul 13;11(7):527. doi: 10.1038/s41419-020-02738-w (PMC7359305; doi:10.1038/s41419-020-02738-w)
Supplement: Supplementary file 1 — Supplementary information [file 41419_2020_2738_MOESM1_ESM.docx]

**CDDIS-19-4740RR**

**SUPPLEMENTARY INFORMATION**

**TABLE OF CONTENTS**

1. **FIGURE LEGENDS**
2. **DATASET LEGENDS**
3. **MATERIALS AND METHODS**
4. **REFERENCES**

**SUPPLEMENTARY FIGURE LEGENDS**

**Fig. S1 - HOTAIRM1 expression and genomic localization. Relative to Figure 1**

**A.** Expression of HOTAIRM1 from 53 non-diseased tissues, according to Genotype-Tissue Expression (GTEx) Analysis Release V7 (dbGaP Accession phs000424.v7.p2). Levels are shown in Transcripts per Million (TPM). Circles represent outliers. Brain tissues are highlighted by a square bracket. C1 spinal cord is pointed by an arrow. Details at [https://gtexportal.org/home/gene/HOTAIRM1#geneExpression](https://gtexportal.org/home/gene/HOTAIRM1%23geneExpression).

**B.** Profiling of markers along iPSC differentiation. qRT-PCR analyses of pluripotency (day 0, *NANOG*), neural stem cells (day 4, *PAX6* and *OLIG2*), neuronal precursor (day 9, *NEUROG2*) and motoneuron (day 16, *CHAT* and *ISLET1*) markers, along ventral spinal lineages differentiation from iPSCs^1^. Differentiation days are reported on the x-axis. For each target, levels are relative to *ATP5O* and expression peaks are set as 1. N=1.

**C.** Schematic representation of human *HOXA* gene cluster. Coding genes are represented as grey boxes, HOTAIRM1 as a black box. Transcription start sites are indicated as arrows.

**Fig. S2 - nHOTAIRM1 signals in purified MNs after RNAse treatment. Relative to Figure 2**

**A.** RNA FISH analysis combined with immunofluorescence in FACS-purified MNs derived from human iPSCs. nHOTAIRM1 in red; *TUBULIN III* (TUB) in green; *ISLET 1/2* (ISLET) in blue.

**B.** The edges of the nuclei are shown in order to highlight the FISH signals inside.

**C.** Localization of nHOTAIRM1 after RNAse treatment. nHOTAIRM1 reduction indicates the specificity of RNA FISH signals.

**D.** Nuclei edges highlighted, as in **B**.

**Fig. S3 - Nucleus/cytoplasm fractionation efficiency in differentiating SH-SY5Y cells knocked down for nHOTAIRM1. Relative to Figure 3**

**A.** qRT-PCR analyses of *pre-GAPDH* transcript and *GAPDH* mRNA in nuclear and cytoplasmic fractions from 3-day RA-treated SH-SY5Y cells, silenced for nHOTAIRM1 by LNA GapmeRs (GapmeR1). Total amounts of *GAPDH* and *pre-GAPDH* in the two compartments are set as 100%. The histogram reports one representative fractionation experiment.

**B.** qRT-PCR analysis of *pre-GAPDH* transcript and *GAPDH* mRNA in nuclear and cytoplasmic fractions from 3-day RA-treated SH-SY5Y cells, silenced for nHOTAIRM1 by siRNAs (siRNA1). Details as in **A**.

**Fig. S4 - Nuclear nHOTAIRM1 regulates *NEUROG2* and its downstream target genes. Relative to Figure 3**

**A.** qRT-PCR analyses of nHOTAIRM1 (left panel) and *NEUROG2* (right panel) upon siRNA-mediated nHOTAIRM1 knockdown (siRNA2) in 3-day RA-treated SH-SY5Y cells. Scrambled siRNAs were used as negative control (CTRL). Data are relative to *GAPDH*. N=4, ** P≤0.01.

**B.** qRT-PCR analyses of nHOTAIRM1 (left panel) and *NEUROG2* (right panel) upon LNA GapmeR-mediated nHOTAIRM1 knockdown (GapmeR2) in 3-day RA-treated SH-SY5Y cells. Scrambled GapmeRs were used as negative control (CTRL). Details as in **A**. N=2 or 3, depending on the sample. * P≤0.05, ** P≤0.01.

**C.** qRT-PCR analyses of *MYCN* upon LNA GapmeR-mediated nHOTAIRM1 knockdown (GapmeR1) in 3-day RA-treated SH-SY5Y cells. Scrambled GapmeRs were used as negative control (CTRL). Details as in **A**. N=2.

**D.** qRT-PCR analyses of *NEUROD* (left panel) and *ASCL1* (right panel) upon siRNA-mediated nHOTAIRM1 knockdown (siRNA2) in 3-day RA-treated SH-SY5Y cells. Scrambled siRNAs were used as negative control (CTRL). Details as in **A**. N=4.

**E.** qRT-PCR analyses of *NEUROD* (left panel) and *ASCL1* (right panel) upon LNA GapmeR-mediated nHOTAIRM1 knockdown (GapmeR2) in 3-day RA-treated SH-SY5Y cells. Scrambled GapmeRs were used as negative control (CTRL). Details as in **A**. N=2, * P≤0.05, ** P≤0.01.

**Fig. S5 - Immunoprecipitation analysis of H3K27me3 on *HPRT1* chromatin in differentiating SH-SY5Y cells and upon nHOTAIRM1 knockdown. Relative to Fig. 4**

**A.** UCSC screenshot showing the chromosomal position and the genomic coordinates of *NEUROG2* locus in the human genome. *NEUROG2* transcriptional start site (TSS) is indicated by an arrow, ChIP target regions located at 400bp (400UP) and 1600bp (1600UP) upstream of *NEUROG2* TSS are boxed.

**B.** UCSC screenshot showing the chromosomal position and the genomic coordinates of *HPRT1* locus in the human genome. *HPRT1* transcriptional start site (TSS) is indicated by an arrow, ChIP target region located at 2000bp (2000UP) upstream of *HPRT1* TSS is boxed.

**C.** H3K27me3 occupancy on *HPRT1* upstream region (HPRT1 2000UP) in differentiating SH-SY5Y cells (day 3), compared to untreated cells (day 0). Enrichments are expressed as percentage relative to Input. N=4.

**D.** H3K27me3 occupancy on *HPRT1* upstream region (HPRT1 2000UP) upon nHOTAIRM1 knockdown by GapmeR1, compared to control transfected cells (CTRL), in 3 day-RA treated SH-SY5Y cells. Details as in **C**.

**Fig. S6 - Expression profile of nHOTAIRM1 in spinal MNs and analysis of U1 RAP assay. Relative to Figure 5**

**A.** qRT-PCR analysis of nHOTAIRM1 expression along spinal MN differentiation of iPSCs. Differentiation days are reported on the x-axis. Expression levels are relative to *ATP5O*, and expression peak (day 5) was set as 1. N=1, 2 or 3, depending on the sample. N=3, * P≤0.05, ** P≤0.01, *** P≤0.001.

**B.** qRT-PCR analysis of U1 RAP assay in differentiating MNs. RNA enrichments over Input, in nHOTAIRM1 or U1 pull-down fractions are reported. Broken axis-histogram allows appreciating low values. Data are expressed as percentage of Input. N=1.

**Fig. S7 - HNRNPK protein and mRNA levels in differentiating SH-SY5Y cells or upon HNRNPK RNAi. Relative to Figure 5**

**A.** Immunoblot analysis of HNRNPK protein along neuronal differentiation of SH-SY5Y cells. Days of RA treatment are reported above each lane. Relative quantity (RQ) is expressed with respect to control cells set as 1 and reported below each lane. HNRNPK levels are quantified relative to GAPDH. N=1.

**B.** qRT-PCR analysis of *HNRNPK* expression upon siRNA-mediated *HNRNPK* knockdown in 3-day RA-treated SH-SY5Y cells. Scrambled siRNAs were used as negative control (CTRL). Data are expressed relative to *GAPDH*. N=4, ** P≤0.01

**Fig. S8 –** **FUS protein and mRNA levels in differentiating SH-SY5Y cells or upon FUS RNAi**. **Relative to Figure 6**

**A.** Immunoblot analysis of FUS protein along neuronal differentiation of SH-SY5Y cells. Days of RA treatment are reported above each lane. Relative quantity (RQ) is expressed with respect to untreated cells set as 1 and reported below each lane. FUS levels are quantified relative to GAPDH. N=1.

**B.** qRT-PCR analysis of *FUS* expression upon siRNA-mediated *FUS* knockdown in 3-day RA-treated SH-SY5Y cells. Scrambled siRNAs were used as negative control (CTRL). Data are expressed relative to *GAPDH*. N=3, *** P≤0.001.

**Fig. S9 – HNRNPK is included in the regulatory cascade mediated by NEUROG2. Relative to Figure 5**

The heterogeneous nuclear protein HNRNPK controls, at the transcription and splicing levels, the expression of the neuronal HOTAIRM1 isoform, which in turn epigenetically regulates the expression of *NEUROG2,* thus affecting the neurogenic pathway.

**DATASET LEGENDS**

**Dataset 1 - Two-way ANOVA and Tukey’s *post hoc* test supported nHOTAIRM1 and NEUROG2 inverse correlation in a specific time-window during iPSC neuronal differentiation**

The dataset includes: 1) a table containing the relative RNA levels of nHOTAIRM1 and *NEUROG2* during neuronal differentiation of iPSCs; 2) a panel describing the two-way ANOVA statistical analysis results; 3) a sheet containing 6 columns: 1) the pairs of comparison among the data; 2) the predicted LS (Least Square) means; 3) the 95% confidence interval of the difference; 4, 5) the significance of each pair of comparisons; 6) the adjusted P values.

**Dataset 2 - *cat*RAPID and RAP-MS analyses revealed nHOTAIRM1 interactors**

The table contains 9 columns: A, B) Protein and Gene Name from Uniprot; C, D) NSAF_HM1, NSAF_U1: averaged NSAF values from the 3 samples, empty if non-present; E, G) observed_HM1, observed_U1: 1 if the protein is present in MS data (0 otherwise); F, H) *cat*RAPID_score_HM1, *cat*RAPID_score_U1: the *cat*RAPID score from omics; I) *cat*RAPID_score_HM1-U1: Difference between F and H. The *cat*RAPID score corresponds to the star rating score. Lines coloured in light red correspond to proteins that were identified experimentally in either HM1 or U1 pull-downs.

**SUPPLEMENTARY MATERIALS AND METHODS**

**Cell cultures and manipulations**

Human neuroblastoma-derived SH-SY5Y cells (ATCC, CRL-2266) were cultured in DMEM-F12 medium (D6421, Sigma-Aldrich) supplemented with 10 % fetal bovine serum (EU Standard, South American Origin, Gibco), 1% sodium pyruvate, 1% L-glutamine and 1% penicillin/streptomycin (Gibco). Cells were induced to differentiation by 10 μM all-*trans*-retinoic acid (RA, R2625, Sigma-Aldrich), for 3 or 6 days.

Specific LNA GapmeRs or siRNAs were lipofected at 100 nM in SH-SY5Y by RNAiMAX reagent (13778030, Invitrogen) in opti-MEM I medium (31985070, Gibco), according to manufacturer’s instructions. Complete growing medium, complemented with RA, was added 5 hours after transfection.

NB4 cell lines were maintained in RPMI 1640 medium supplemented with 1x penicillin/streptomycin solution, 1x L-glutamine, and 10% fetal bovine serum and were induced to granulocytic differentiation by 1 μM RA treatment.

IPSCs differentiation^2^ was carried out in N2B27 medium supplemented with 1 μM RA and 1 μM SAG (566660, Merck Millipore) for 12 days, in the presence of 10 μM SB431542 (130-106-275, Miltenyi Biotec) and 100 nM LDN-193189 (130-106-540, Miltenyi Biotec) from day 0 to 6, and 5 μM DAPT (D5942, Sigma-Aldrich) and 4 μM SU-5402 (SML0443-5MG, Sigma-Aldrich) from day 6 to 12. Cells were sorted at day 12-13 using a FACSAria III (BD Biosciences) and re-plated on poly-L-ornithine- and laminin- coated dishes (both from Sigma-Aldrich) in Neural Medium.

Human spinal MNs were obtained from iPSC-NIL cells^3^ with the protocol detailed in^4^. iPSC-NIL cells were dissociated to single cells with Accutase (A6964-100ML, Thermo Fisher Scientific) and plated in Nutristem-XF/FF medium (05-100-1A, Biological Industries) supplemented with 10 μM Y-27632 (ROCK inhibitor) (ALX-270-333-M005, Enzo Life Sciences) on Matrigel (BD Biosciences). Differentiation was induced by adding 1 μg/ml doxycycline (Thermo Fisher Scientific) in DMEM/F12 (D6421-500ML, Sigma Aldrich). After 48 hours-induction, medium was changed to Neurobasal medium (21103049, Thermo Fisher Scientific) supplemented with 1X B27 (17504, Gibco), 1X Glutamax (35050038, Thermo Fisher Scientific), 1X NEAA (11140035, Thermo Fisher Scientific) and 0.5X Penicillin/Streptomycin (P4333-100ML, Sigma Aldrich), containing 5 μM DAPT and 4 μM SU5402.

### RNA FISH, RNA/DNA FISH and Immunofluorescence

Cell were cultured on glass coverslips precoated with 0,4 mg/ml Collagen Rat Tail and 0,01% poly-L-ornithine/Murine Laminin 20μg/ml. Cell fixation was carried out in 4% paraformaldehyde/PBS (Electron Microscopy Sciences, Hatfield, PA) for 20 min at 4°C.

### To visualize nHOTAIRM1, RNA FISH was performed by using synthetic DNA oligonucleotides (Biotin 3’-end conjugated) detected with anti-Biotin Cy3 conjugated (Jackson ImmunoResearch, 200-162-211). A quality control was obtained by performing the hybridization on sample pre-treated with a mixture of ribonucleases containing RNAse A 200 μg/ml (Sigma, R6513), RNAse DNAse free 100 μg/ml (Roche, 11119915001) and SUPERase in 250U/ml (Invitrogen, AM2694) for 1h at 37°C.

### DNA FISH to detect *NEUROG2* genomic locus (BAC clone RP11-806L21) was performed according to^5^. To combine DNA FISH protocol with RNA FISH, a sequential approach was applied. In brief, RNA FISH was initially performed and recorded, followed by DNA. The same confocal fields were acquired to merge the RNA and DNA FISH signals at the same nuclear coordinates. For FISH/Immunofluorescence analyses, cells were stained for EZH2 AC22 (Cell Signaling, 1:50), anti-beta Tubulin III (anti-Tuj 1) (Sigma T2200, 1:100) and anti-Islet 1/2 39.4D5 (DSHB, 1:50) in 1% BSA/TN Buffer and incubated overnight at 4 °C. Secondary antibodies were used to detect the primary antibodies: goat anti-rabbit Alexafluor 488-conjugated (ThermoFisher A-11008; 1:300), goat anti-mouse Alexafluor 488-conjugated (ThermoFisher A-11001) and donkey anti-mouse Alexa Fluor 647-conjugated (Invitrogen A-31571; 1:100) in 1% goat serum/1% donkey serum/TN buffer for 45 minutes at room temperature. After washings with PBS, the coverslips were incubated with DAPI solution (Sigma, D9542; 1μg/ml/PBS) for 5 minutes at room temperature and then mounted using ProLong Diamond Antifade Mountant (ThermoFisher Scientific, P-36961). Samples were imaged with 60X NA1.35 oil (UPLANSApo) objective on confocal microscopes spinning disk Olympus IX73, equipped with CoolSNAP Myo CCD camera (Photometrics) and a Lumencor Spectra X LED illumination. The images were collected as Z stacks with a Z step size of 0.2 μm at 1024 x 1024 pixels. Intensity balance of the signal/background was determined by using MetaMorph (Molecular Devices) and FIJI software to the entire image.

### Processing and post-acquisition quantifications on RNA/DNA FISH and Immunofluorescence images were performed according to^5^. In particular, spatial proximity analysis of RNA FISH and DNA FISH signals was performed by computing through FIJI software the 2D spots-distance on Z-projection images (Nearest-Neighbours analysis). Unpaired, paired or overlapped spatial conditions were represented as a dimensional value (Normalized Distances, ND) to take into account the nuclei shapes (ND= Interallelic Distances/d, d=major axis+minor axis/2). The percentage of NEUROG2 loci that show spatial proximity with nHOTAIRM1 spots was represented as mean ± SEM of two technical replicates on 96 nuclei analysed. The co-planarity of the RNA FISH and immunofluorescence signals (nHOTAIRM1/EZH2 co-staining) was assessed based on the co-existence of maximum fluorescence values on the same Z-plane. 3D-processing and quantification of colocalized signalswere performed with FIJI software by using 3D viewer and ComDet 0.3.7 plugin on 39 nuclei of SH-SY5Y cells at day 6 of differentiation. The percentage of nHOTAIRM1 signals that show colocalization with EZH2 protein was represented as mean ± SEM of three biological replicates.

**RNA extraction and analysis**

Total RNA was extracted by Direct-zol RNA MiniPrep (R2052, Zymo Research). For quantitative real-time PCR (qRT-PCR) assay, cDNA was synthetized by Takara PrimeScript RT Reagent Kit (RR037A, Takara-bio). qPCR detection was performed using PowerUp SYBR Green Master Mix (A25742, Life Technologies) on a 7500 Fast Real-Time PCR (Applied Biosystem). RNA expression, relative to GAPDH or ATP5O used as reference targets, was analysed through the 2^-ΔΔCt^ (Livak) Method.

For full-length isoform determination, cDNAs were analysed by semi-quantitative reverse transcriptase-PCR (RT-PCR), using specific oligonucleotides. Amplification reactions were collected, agarose-separated and fractionation patterns were revealed by ChemiDoc XRS+ Molecular Imager (Bio-Rad).

To check for nHOTAIRM1 polyadenilation, cDNAs were obtained using SuperScript II Reverse Transcriptase (18064014, Invitrogen) in the presence of oligo dT or random hexamers separately. cDNAs were analysed by qRT-PCR and compared to *GAPDH* or U16 RNA, used as poly(A) plus or poly(A) minus reference species, respectively.

**Immunoblotting**

### Protein samples for immunoblotting were collected in RIPA Buffer (50 mM Tris–HCl [pH 8], 150 mM EGTA, 150 mM NaCl, 50 mM NaF, 10 % glycerol, 1.5 mM MgCl2, 1 % Triton).

### They were separated on gradient poly-acrylamide gels and transferred to Amersham Protran 0.45 um nitrocellulose membrane (GE Healthcare Life Sciences), through the NuPAGE System (Invitrogen). Immunoblots were incubated with antibodies from Santa Cruz Biotechnology (anti-GAPDH, sc-32233; anti-HNRNPK, sc-28380; anti-FUS, sc-47711) or Abcam (anti-NEUROG2, ab109236 and anti-SUZ12, ab12073).

### Staining was performed by WesternBright ECL (K-12045-D50, Advansta) detected by ChemiDoc XRS+ Molecular Imager (Bio-Rad) and quantified through the Image Lab Software (release 3.0.1).

**RNA Immunoprecipitation (RIP) Assay**

50x10^6 SH-SY5Y cells were grown with RA for 3 days. Cell nuclei were isolated in Nuclear Isolation Buffer in PBS (0.25 M sucrose, 8 mM Tris–HCl pH 7.5, 4 mM MgCl_2_, 0.8 % Triton X-100) and disrupted in RIP buffer (150 mM KCl, 25 mM Tris pH 7.4, 5 mM EDTA, 0.5 mM DTT, 0.5 % NP40 1× PIC, 1 mM PMSF, 40U/ml RNAse inhibitor). 2mg of nuclear extracts were incubated with 10μg of SUZ12 antibody (ab12073, Abcam) or IgG (normal rabbit IgG sc-2027, Santa Cruz Biotechnology) as control and precipitated through ProteinG-Dynabeads resin (10004D, Invitrogen). Immunoprecipitated proteins were prepared in RIPA buffer for immunoblot. Immunoprecipitated RNA was extracted by Direct-zol RNA MiniPrep (Zymo Research), retro-transcribed by Superscript Vilo cDNA synthesis Kit (11754050, Invitrogen) and analysed by qRT-PCR using specific primers. RNA enrichments were reported as percentage of the input.

**Chromatin Immunoprecipitation (ChIP) assay**

Immunoprecipitation was performed using the MAGnify Chromatin Immunoprecipitation System kit (492024, Invitrogen) according to the manufacturer's protocol. Briefly, chromatin extracts were sonicated and immunoprecipitated with 5 μg of mouse anti-Histone H3 (tri-methyl K27) antibody (mAbcam 6002), or IgG antibodies (normal mouse IgG sc-2025, Santa Cruz Biotechnology) according to the manufacturer's protocol (MAGnify Chromatin Immunoprecipitation System kit, 492024, Invitrogen).

A standard curve was generated for each primer pair testing 5-point dilutions of input sample. NEUROG2 promoter fold enrichment was quantified using qRT-PCR (SYBR Green, QIAGEN) and calculated as a percentage of Input chromatin (% Inp) after background (IgG) subtraction. Data from control *vs* LNA GapmeRs conditions were normalized to HPRT1 upstream region. N=3.

**Crosslinking Immunoprecipitation (CLIP) assay**

Cells were collected in NP-40 lysis buffer (50 mM HEPES-KOH, 150 mM KCl, 2 mM EDTA, 1 mM NaF, 0.5% (v/v) NP40 pH 7.4, 0.5mM DTT) by low sonication and centrifugation at 20.000 rcf. For ribonucleoprotein immunoprecipitation, 2mg of cytoplasmic extracts were incubated with 10 μg of FUS antibody (sc-47711) or IgG as a control, and coupled to ProteinG Dynabeads resin (10004D, Invitrogen). After washing with high-salt buffer (50mM HEPES-KOH, pH7.5, 500mM KCl, 0.05%(v/v) NP40, 0.5mM DTT), immunoprecipitated proteins were collected in RIPA buffer and analysed by immunoblotting. Immunoprecipitated RNA was treated by Proteinase K, extracted by Direct-zol RNA MiniPrep (Zymo Research), retro-transcribed by Superscript Vilo cDNA synthesis Kit (11754050, Invitrogen) and analysed by qRT-PCR using specific primers. RNA enrichments were reported as percentage of the input.

**RNA Antisense Purification-Mass Spectrometry (RAP-MS) assay**

90nt-long probes, antisense to nHOTAIRM1 or U1 snRNA, were designed to cover their entire sequence without any overlap. According to BLAT tool oligonucleotides with 30bp perfect matches to any expressed off-target were excluded. RNA secondary structures predictions (RNAfold) were used to select probes aligning to unfolded RNA region. 5’-biotin modified DNA primers were synthesized by Integrated DNA Technologies.

20x10^6 iPSCs were differentiated for 4 days and underwent RAP assay according to^6^ (with minor modifications). Briefly, cells were UV-crosslinked at 8000×100 µj/cm^2^ (energy), collected in Total Cell Lysis buffer (10mM Tris-HCl, Ph 7.5, 500mM LiCl, 0.5% DDM, 0.2% SDS, 0.1% sodium deoxycholate) and sheared. Lysate was sonicated, was made 2M Urea and 1.25mM DTT and precleared. Extract was then incubated at 67**°**C for 2 hours with biotinylated antisense probes (10μg), specific for nHotairM1 or U1 RNA, before adding streptavidin-coated beads (Promega, Z5481). After extensive bead washing, RNA was eluted through NLS elution buffer (20mM Tris HCl pH 8, 10mM EDTA, 2% NLS, 2.5mM DTT) for enrichment analysis by qRT-PCR, whereas proteins were eluted by Benzonase Elution buffer (20mM Tris HCl pH 8, 0.05% NLS, 2mM MgCl2, 0.5 mN DTT) for MS analysis. TCA-precipitated proteins were analysed by MS through Orbitrap ELITE/C18 Accucore 50cm at the Plateforme de Protéomique, IGBMC (Illkirch, France). Spectral counts were accounted through a Normalized Spectrum Abundance Factor (NSAF) score.

***cat*RAPID analyses**

The catRAPID algorithm predicts the interaction potential of a protein and RNA pair through their van der Waals, hydrogen bonding and secondary structure properties.

In our calculations of nHOTAIRM1 and U1 interactions we used the human library of proteins (3500 proteins) available in the *cat*RAPID omics server (<http://s.tartaglialab.com/page/catrapid_omics_group>)^7^. As positives, we considered all the interaction partners with a significant NSAF (normalized spectrum abundance factor) score > 0.01. Proteins abundant in brain (<https://pax-db.org/dataset/9606/180>) but not detected in RAP-MS experiments were considered as negative. HNRNPK and FUS have scores >2 that are 3 standard deviations above the average interaction of human RNA-binding proteins (mean=1.15; standard deviation=0.30).

**Statistical analyses**

Data shown in histograms are the mean ±SEM from two to four biological replicates. In specific cases single experiments were analysed (N is indicated in Figure Legends). The Standard Error of the Mean (SEM) was calculated for the normalized mean values from qRT-PCR (relative quantities) and then opportunely propagated for the fold change representation. Statistical significance was determined by 2-tailed paired Student's t-test (performed with GraphPad QuickCalcs) which does not assume that the two sets of data are sampled from populations with equal variances. A p value (P)<0.05 was considered as significant.

As for Figure 1A, analyses of variance between nHOTAIRM1 and *NEUROG2* RNA levels were done using two-way ANOVA, performed with GraphPad Prism 8.0 (GraphPad Software Inc.). To account for the variations shown, *post hoc* Tukey’s test (multiple comparison procedure) was performed to compare the nHOTAIRM1 and *NEUROG2* RNA levels at corresponding time points, and each RNA levels across the days of differentiation.

**SUPPLEMENTARY REFERENCES**

1. Lenzi, J. et al. ALS mutant FUS proteins are recruited into stress granules in induced Pluripotent Stem Cells (iPSCs) derived motoneurons. *Dis Model Mech* **8**, 755-66 (2015).
2. De Santis, R. et al. FUS Mutant Human Motoneurons Display Altered Transcriptome and microRNA Pathways with Implications for ALS Pathogenesis. *Stem Cell Reports* **9**, 1450–1462 (2017).
3. De Santis, R. et al. Direct conversion of human pluripotent stem cells into cranial motor neurons using a piggyBac vector. *Stem Cell Research* **29**, 189–196 (2018).
4. Garone, M.G. et al. Conversion of Human Induced Pluripotent Stem Cells (iPSCs) into Functional Spinal and Cranial Motor Neurons Using PiggyBac Vectors. *J Vis Exp* **147** (2019).
5. Ballarino, M. et al. Deficiency in the nuclear long noncoding RNA Charme causes myogenic defects and heart remodeling in mice. *EMBO J* **37**, e99697 (2018).
6. McHugh, C.A. & Guttman, M. RAP-MS: A Method to Identify Proteins that Interact Directly with a Specific RNA Molecule in Cells. *Methods Mol Biol* **1649**, 473-488 (2018).
7. Agostini, F. et al. catRAPID omics: a web server for large-scale prediction of protein-RNA interactions. *Bioinformatics* **29**, 2928-30 (2013).
