# Supplementary figures and images for: HOTAIRM1 regulates neuronal differentiation by modulating NEUROGENIN 2 and the downstream neurogenic cascade
Source: Cell Death Dis. 2020 Jul 13;11(7):527. doi: 10.1038/s41419-020-02738-w (PMC7359305; doi:10.1038/s41419-020-02738-w)

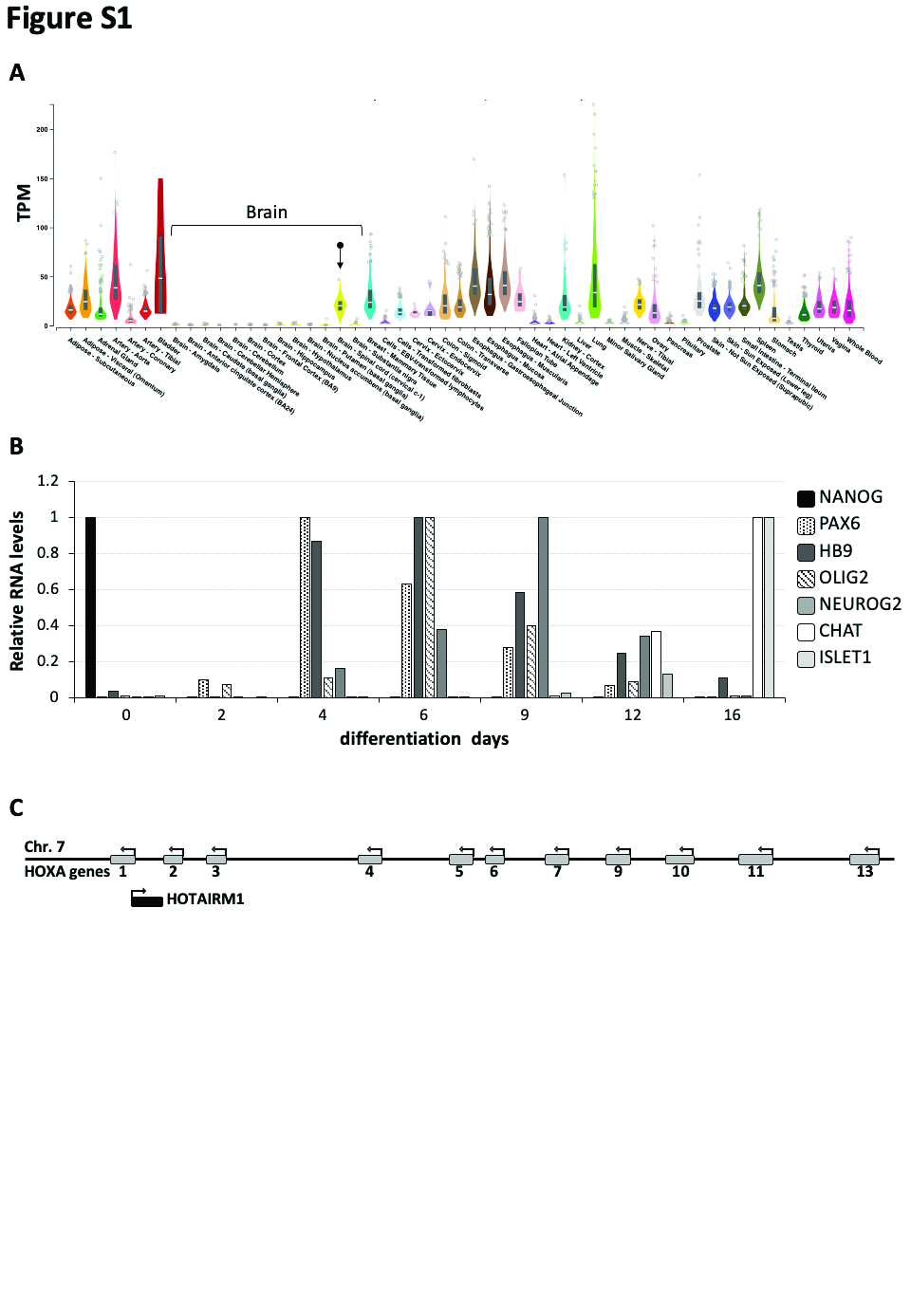

Supplement: Supplementary file 2 — Supplementary figure 1 [file 41419_2020_2738_MOESM2_ESM.tif]

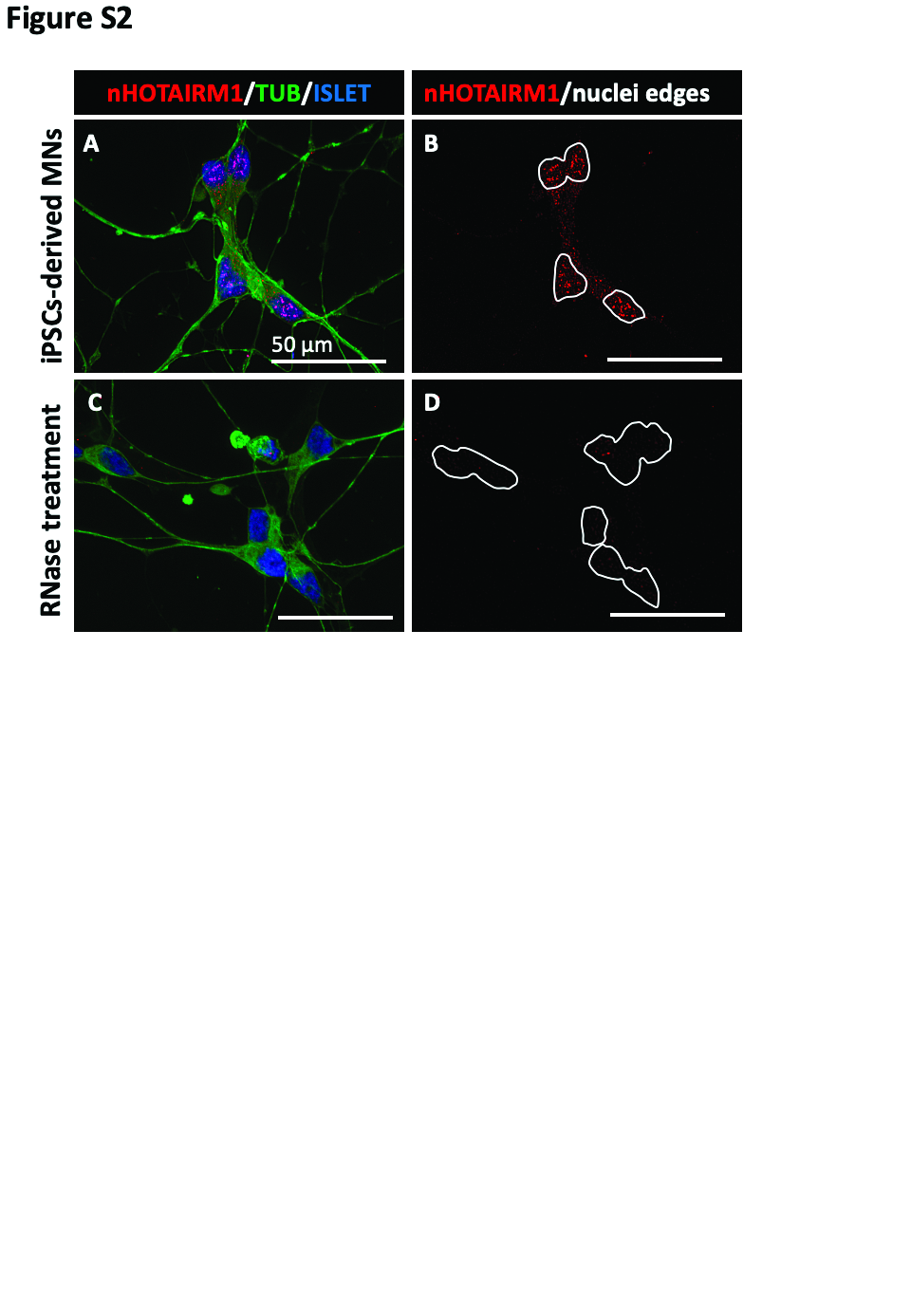

Supplement: Supplementary file 3 — Supplementary figure 2 [file 41419_2020_2738_MOESM3_ESM.tif]

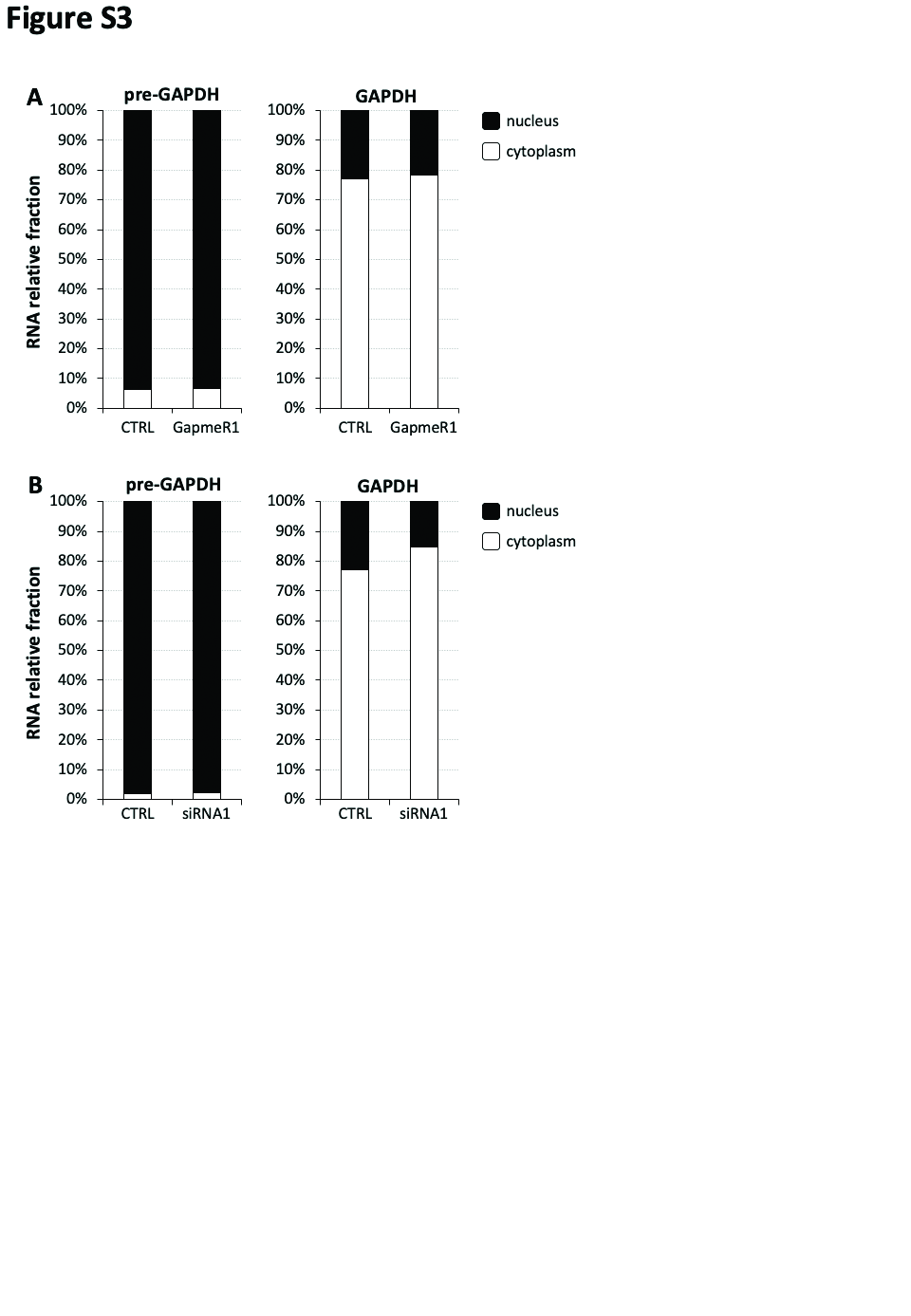

Supplement: Supplementary file 4 — Supplementary figure 3 [file 41419_2020_2738_MOESM4_ESM.tif]

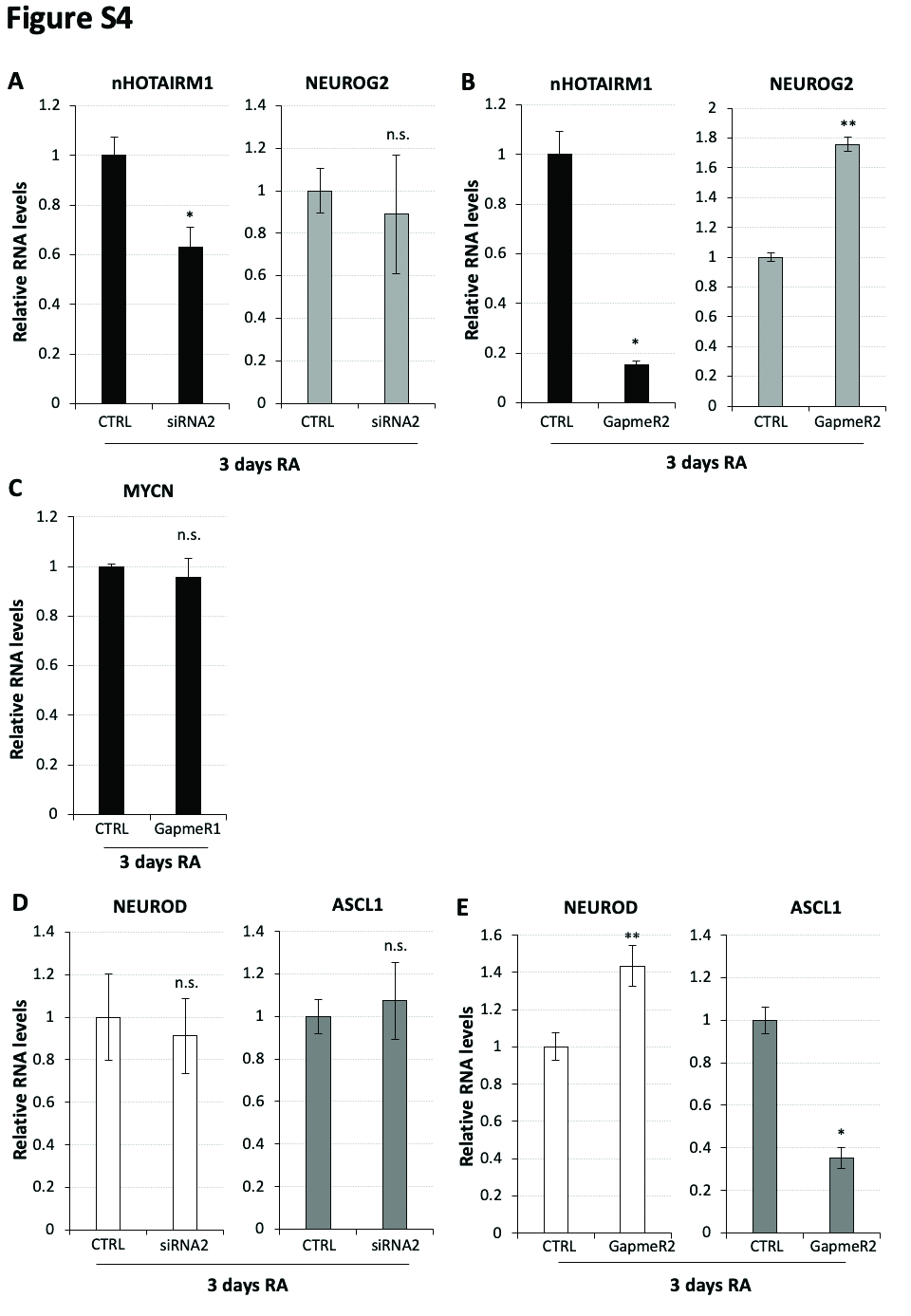

Supplement: Supplementary file 5 — Supplementary figure 4 [file 41419_2020_2738_MOESM5_ESM.tif]

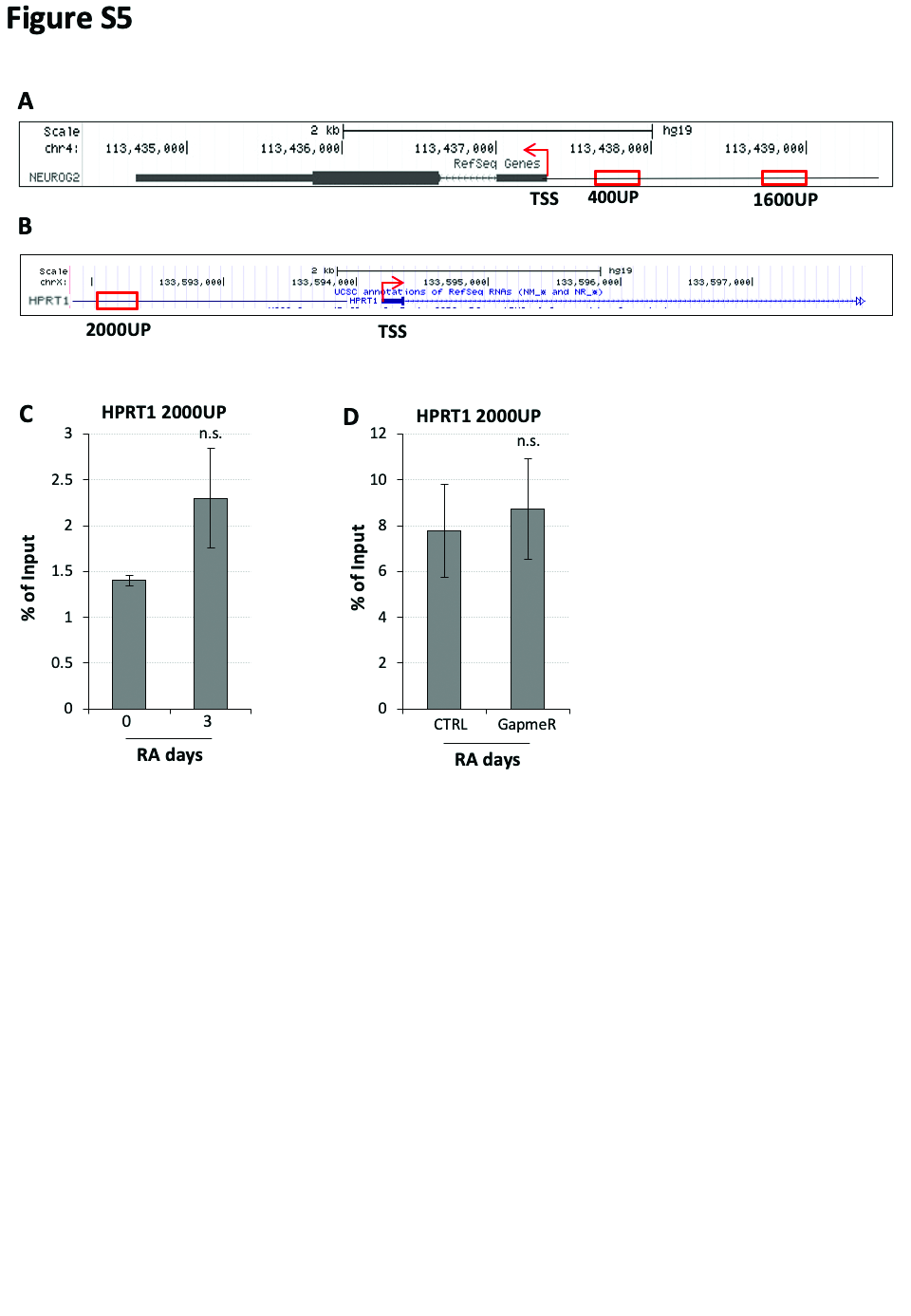

Supplement: Supplementary file 6 — Supplementary figure 5 [file 41419_2020_2738_MOESM6_ESM.tif]

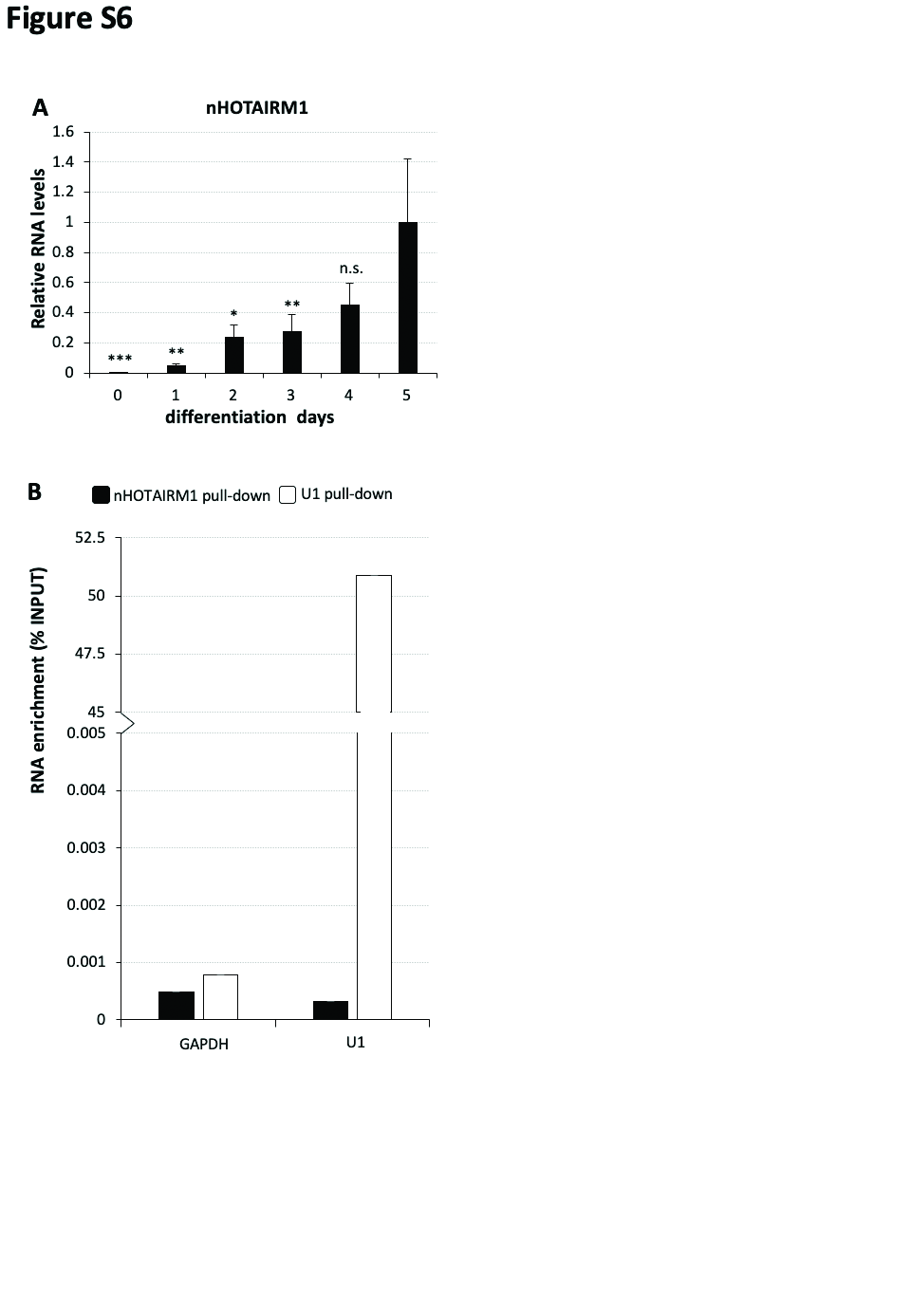

Supplement: Supplementary file 7 — Supplementary figure 6 [file 41419_2020_2738_MOESM7_ESM.tif]

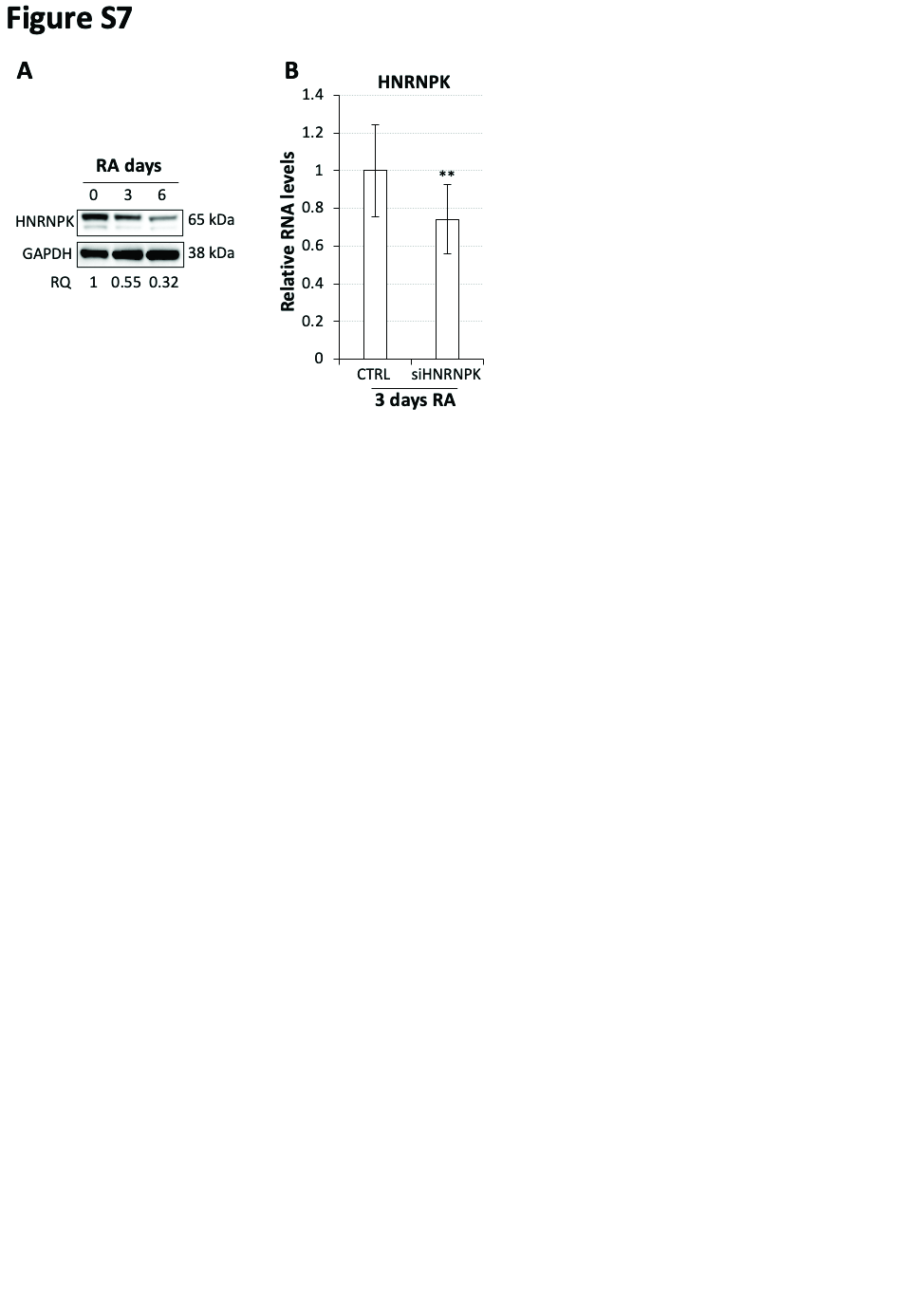

Supplement: Supplementary file 8 — Supplementary figure 7 [file 41419_2020_2738_MOESM8_ESM.tif]

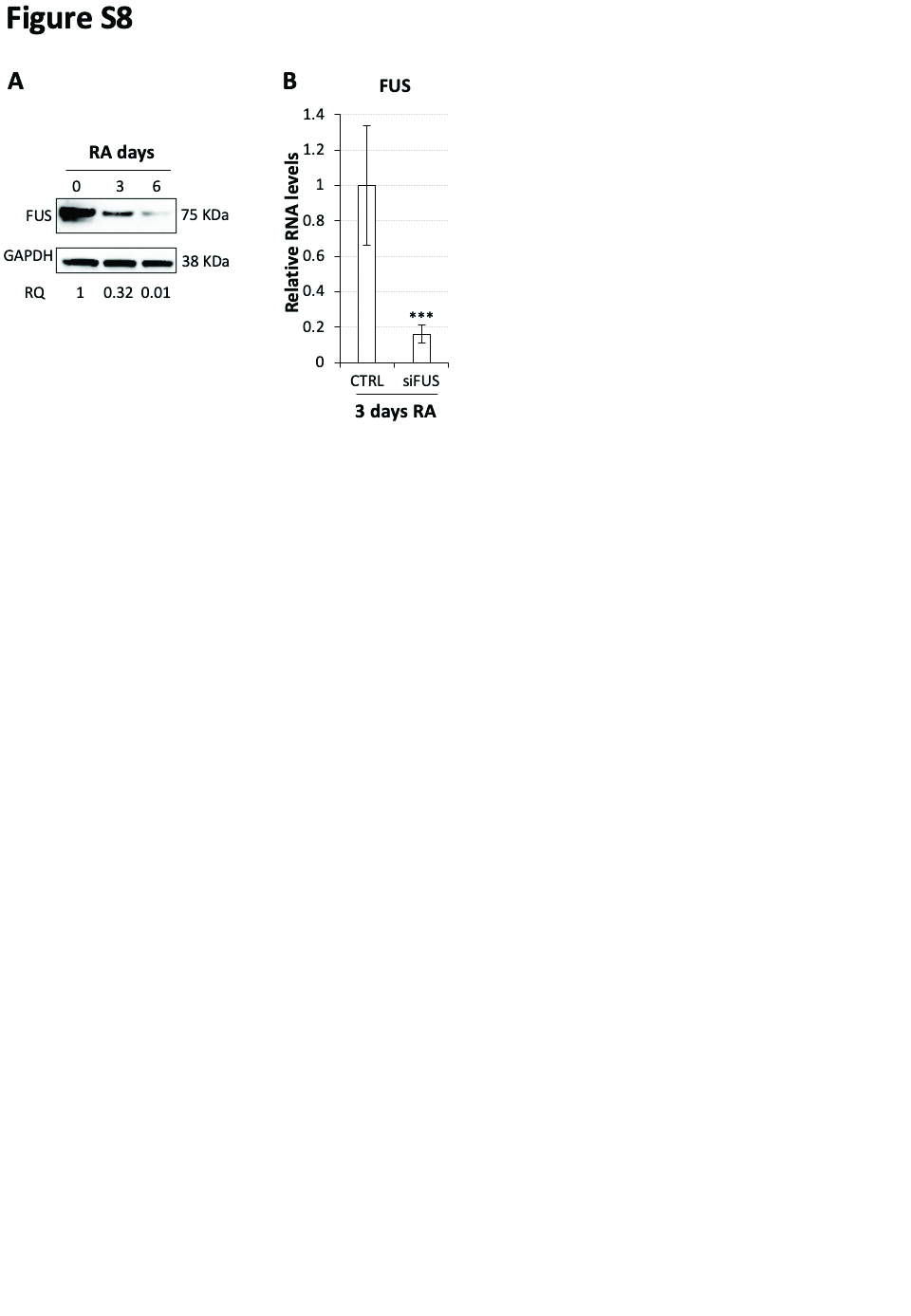

Supplement: Supplementary file 9 — Supplementary figure 8 [file 41419_2020_2738_MOESM9_ESM.tif]

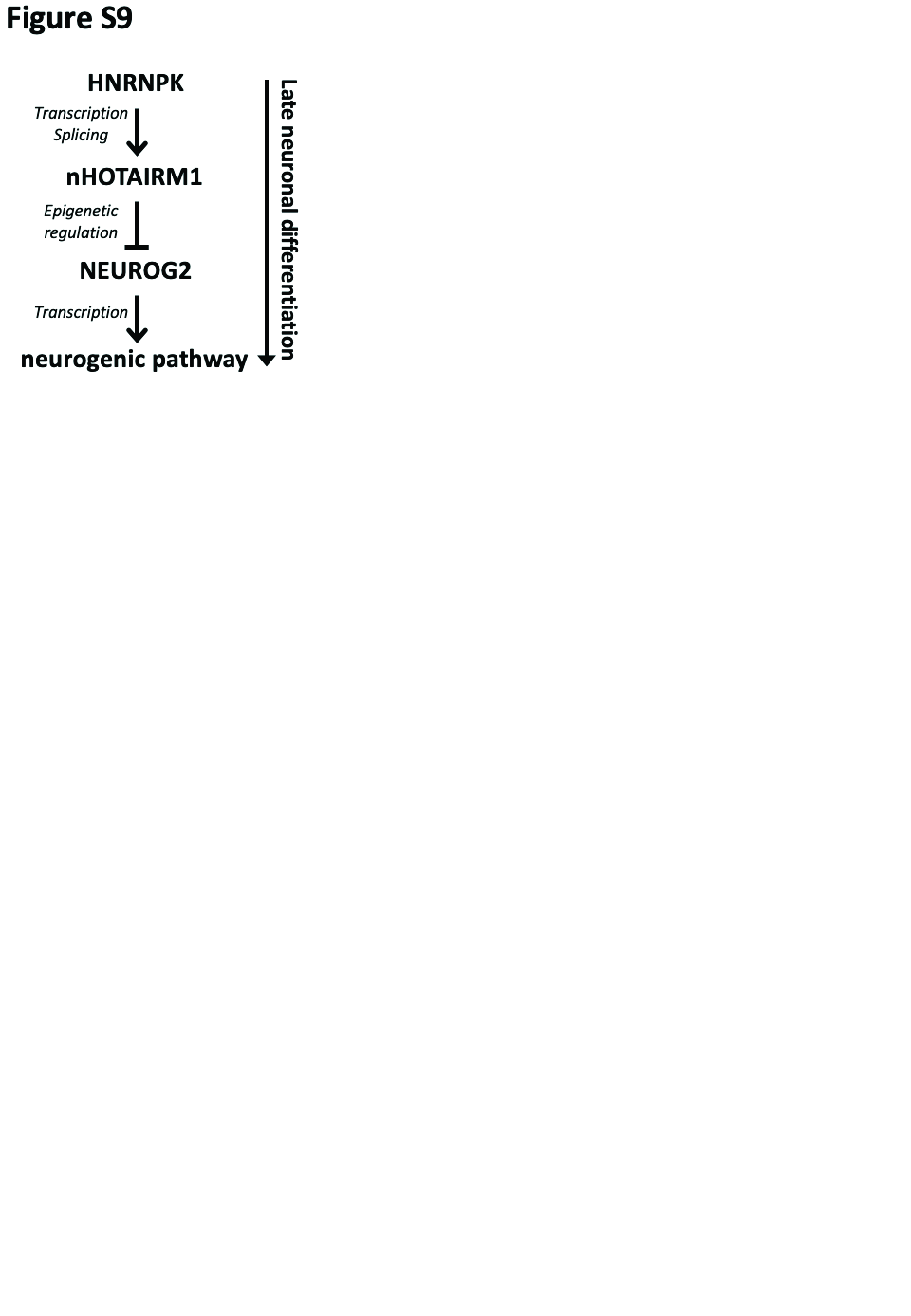

Supplement: Supplementary file 10 — Supplementary figure 9 [file 41419_2020_2738_MOESM10_ESM.tif]
